# Effects of Ocean Acidification on Temperate Coastal Marine Ecosystems and Fisheries in the Northeast Pacific

**DOI:** 10.1371/journal.pone.0117533

**Published:** 2015-02-11

**Authors:** Rowan Haigh, Debby Ianson, Carrie A. Holt, Holly E. Neate, Andrew M. Edwards

**Affiliations:** 1 Pacific Biological Station, Fisheries and Oceans Canada, 3190 Hammond Bay Road, Nanaimo, British Columbia, V9T 6N7, Canada; 2 Institute of Ocean Sciences, Fisheries and Oceans Canada, 9860 West Saanich Road, Sidney, British Columbia, V8L 4B2, Canada; 3 Department of Biology, University of Victoria, P.O. Box 1700, Station CSC, Victoria, British Columbia, V8W 2Y2, Canada; University of Hong Kong, HONG KONG

## Abstract

As the oceans absorb anthropogenic CO_2_ they become more acidic, a problem termed *ocean acidification* (OA). Since this increase in CO_2_ is occurring rapidly, OA may have profound implications for marine ecosystems. In the temperate northeast Pacific, fisheries play key economic and cultural roles and provide significant employment, especially in rural areas. In British Columbia (BC), sport (recreational) fishing generates more income than commercial fishing (including the expanding aquaculture industry). Salmon (fished recreationally and farmed) and Pacific Halibut are responsible for the majority of fishery-related income. This region naturally has relatively acidic (low pH) waters due to ocean circulation, and so may be particularly vulnerable to OA. We have analyzed available data to provide a current description of the marine ecosystem, focusing on vertical distributions of commercially harvested groups in BC in the context of local carbon and pH conditions. We then evaluated the potential impact of OA on this temperate marine system using currently available studies. Our results highlight significant knowledge gaps. Above trophic levels 2–3 (where most local fishery-income is generated), little is known about the direct impact of OA, and more importantly about the combined impact of multi-stressors, like temperature, that are also changing as our climate changes. There is evidence that OA may have indirect negative impacts on finfish through changes at lower trophic levels and in habitats. In particular, OA may lead to increased fish-killing algal blooms that can affect the lucrative salmon aquaculture industry. On the other hand, some species of locally farmed shellfish have been well-studied and exhibit significant negative direct impacts associated with OA, especially at the larval stage. We summarize the direct and indirect impacts of OA on all groups of marine organisms in this region and provide conclusions, ordered by immediacy and certainty.

## Introduction

Fossil fuel burning and changes in land use by humankind have increased atmospheric carbon dioxide (CO_2_) at an unprecedented rate, causing our climate to change [1]. A significant portion of this anthropogenic CO_2_ (∼30%; [2]) has been absorbed by the ocean. When CO_2_ enters the ocean it combines with water (H_2_O), resulting in an increase in the concentration of hydrogen ions [H^+^] and an increase in acidity (decrease in pH [3, 4]. Therefore, as our climate changes, our oceans become more acidic due to anthropogenic contributions, a problem termed Ocean Acidification (OA) [5].

While anthropogenic atmospheric CO_2_ dominates contributions to OA on a global scale, other anthropogenic sources may be significant on a local scale [6]. For example, acid rain from vehicle emissions and industry cause an increase in ocean acidity, which is likely relevant, at least near (and downwind of) urbanized regions [7]. Any addition of organic carbon to the ocean, such as sewage, decomposes to dissolved inorganic carbon (DIC), and increases acidity. Agricultural run-off provides nutrients which then fuel (an anthropogenic) increase in production of organic carbon in the ocean [8], again increasing acidity.

Aquatic acidity is most commonly reported as pH. However, pH is difficult to determine accurately in saltwater because of the additional ions present in solution [9]. It is closely linked with carbonate chemistry in the ocean, which is complex. To quantify the *carbon state* (*i.e.* the concentration of each chemical form of DIC present) in seawater, two of four measured parameters—DIC, pH, total alkalinity (TA), and partial pressure of CO_2_ (*P*_CO2_)—must be known, in addition to temperature and salinity. To be more accurate, phosphate and silicic acid concentrations are also required [10]. In the past, pH has most often been determined from DIC and TA (*e.g.* [11]). (TA is the acid neutralizing capacity of the solution, which is not simply related to pH in seawater [10].) Thus, although one can generalize to say that high DIC is usually associated with low pH (or high *P*_CO2_), more information, *e.g.* TA, is required to be quantitative.

The carbon state is relevant to biology. Most of the DIC in the ocean occurs in the form of bicarbonate (${\mathrm{HCO}}_{3}^{-}$) and carbonate (${\text{CO}}_{3}^{2-}$), with less than 1% in the form of CO_2_. When pH decreases, the balance between ${\mathrm{HCO}}_{3}^{-}$ and ${\text{CO}}_{3}^{2-}$ changes so that there is less ${\text{CO}}_{3}^{2-}$. This shift has important implications for plants and animals that build calcium carbonate (CaCO_3_ structures (*e.g.* shellfish, corals) [12]. Two mineral forms of CaCO_3_ (aragonite and calcite) are common in biological structures. The aragonitic form is more soluble than calcite given the same environmental conditions [13]; therefore, creatures that use aragonite are more susceptible to OA than those that use calcite [12]. The ease with which these minerals are formed is quantified by the saturation state (Ω), such that as Ω decreases, dissolution increases [14]. The water is *undersaturated* with respect to CaCO_3_ when the chemical rate of dissolution exceeds the rate of formation [15]. For organisms that precipitate CaCO_3_, decreasing Ω means that more energy is required to build and maintain their carbonate structures [16, 17].

Marine organisms are also affected by carbon state (defined above) and OA in other ways. All marine animals need to rid themselves of metabolically produced CO_2_ through respiration. The effectiveness of this removal is dependent, in part, on the ambient *P*_CO2_ of the medium (*e.g.* [18]). Similarly, plants and animals rely on pH to regulate ion transport, and the energy they must expend to maintain intra- and extracellular pH depends on ambient pH (*e.g.* [19]). Thus, there is no one carbon parameter that best indicates OA impacts on all marine organisms, and so full knowledge of the complete carbon state is desirable (*e.g.* [20]).

A large and growing number of studies have been undertaken regarding OA (S1 Table). To understand and predict biological impacts, an increasing number of experiments have been completed that attempt to emulate future ocean conditions in the laboratory. Experimental conditions are usually defined by controlling either the *P*_CO2_ or the pH (*e.g.* S2 Table) and recently an internationally accepted guide has been published that describes the techniques used [21]. In most of these experiments, present-day conditions (the control) are set at either atmospheric *P*_CO2_ (∼400 *μ*atm at the time of writing) or the estimated current global average pH of the surface ocean, which is 8.1 [5]. However, marine organisms in the natural environment may experience values that are significantly different depending on location and the depth that they occupy.

In the ocean, DIC (and *P*_CO2_) generally increase with depth while pH decreases. In other words, low pH conditions naturally occur at depth. This partitioning of inorganic carbon towards deeper parts of the ocean is due in large part to the ‘biological pump’ that allows the ocean to hold more carbon [22]. Photosynthesis in the surface draws down DIC (which increases pH) and produces organic forms of carbon. Some of this organic carbon falls to deeper levels, where it decays back to DIC (decreasing pH).

### British Columbia—oceanography

**Fig 1:**
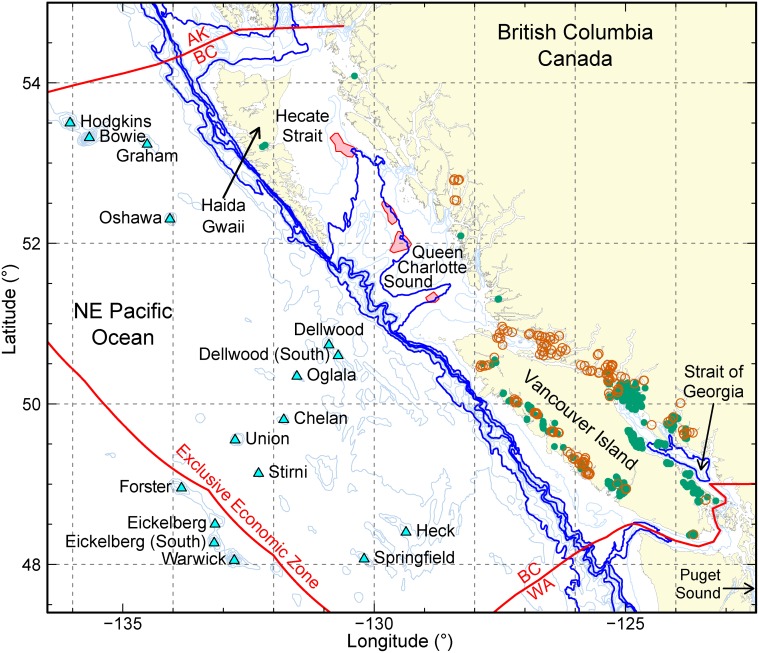
British Columbia (BC) coastline and bathymetry (isobaths in metres: thin grey—100, 200, 300, …, 1000, 1250, 1500, 2000, 2500; thick blue—200, 500, 800, and 1600). The continental slope along most of BC comprises steep slopes, especially along the west coasts of Haida Gwaii and northern Vancouver Island. Hecate Strait is largely dominated by shallow waters and a flat seafloor. Sponge reef core protected areas in Hecate Strait and Queen Charlotte Sound are shaded pink. The Strait of Georgia forms a large inland sea that is heavily influenced by river runoff and tidal currents. Saltwater finfish farm and hatchery sites are indicated by open red circles, commercial marine shellfish farms are indicated by solid green circles [345]. Select seamounts [346] are marked by blue triangles. Canada’s Exclusive Economic Zone (200-nautical miles offshore) is delimited in red. Map was prepared using PBSmapping in R [347]. The R code is provided as Supporting Information (S1 Code).

**Fig 2:**
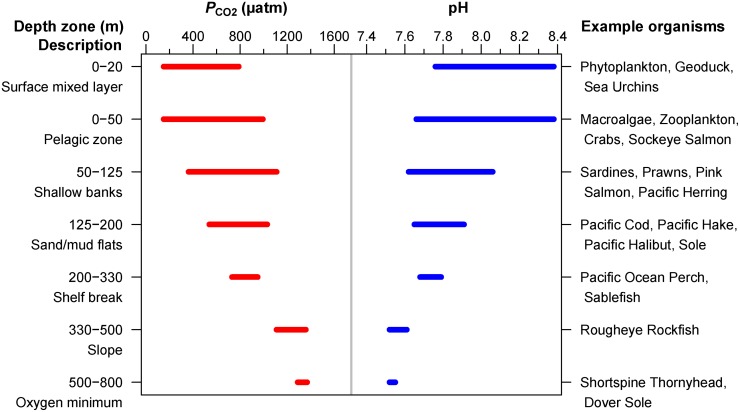
Estimated present-day ranges of *P*_CO2_ (red) and pH (blue) during spring [40] and summer [27] for various depth zones along the outer BC continental shelf, with typical species found in each zone (see Methods). There are numerous data above 50m and few below 125 m. The number of values in each depth zone from top to bottom are: 70, 116, 33, 45, 5, 4 and 2, respectively. Above 50 m, the distributions of values are skewed, such that high *P*_CO2_ (low pH) extremes occur less often than the low *P*_CO2_ (high pH) extremes. Data and R code for this figure are provided as Supporting Information (S2 Code).

British Columbia (BC) makes up 27,000 km (17,000 mi) of the temperate northeast Pacific coastline. Circulation along this coast (Fig. 1) is dynamic so that large changes in carbon parameters occur both in space (*e.g.* [23]) and time (*e.g.* [24, 25]). Coastal upwelling along the west coast of Vancouver Island (WCVI) [26] brings subsurface water high in DIC into the surface mixed layer [27] so that low pH (*e.g.* 7.6) is found at relatively shallow depths, *e.g.* above 125 m (Fig. 2). Furthermore, these subsurface waters are enriched in DIC relative to waters at the same depth in other ocean basins, simply because north Pacific water is relatively ‘old’ and has had more time to receive organic matter [28, 29]. Upwelled waters are also rich in nutrients that are limiting to phytoplankton growth and so cause high primary production that increases pH at times. In fact, the WCVI enjoys the highest productivity of any zone on the northeast Pacific coast [30]. Consequently, present-day ranges in pH in the surface mixed layer along the outer BC coast span a remarkable range (7.8–8.4; Fig. 2). The low end of this range is significantly lower than the benchmark of present-day average global surface ocean pH (8.1).

In protected waters (*e.g.* Strait of Georgia, Fig. 1) less data are available relative to the WCVI. These data show similar (or larger) ranges in surface pH and *P*_CO2_ (unpublished data, DI), which are also similar to values found just to the south in the protected waters of Puget Sound, Washington State (WA) [6, 31]. Again, a critical feature in these waterways is low surface pH (high *P*_CO2_) relative to global averages, especially during the winter season [32].

### British Columbia—fishery

Fisheries and aquaculture play an important role in the BC economy, contributing over $650 million (we quote all dollar values in Canadian dollars) to the provincial gross domestic product (GDP) in 2011 [33]. Sport (or recreational) fishing, mainly for salmon and Pacific Halibut, is responsible for approximately 50% of this contribution, while the wild (or capture) fishery makes up ∼15% and aquaculture ∼10%. Marine ecosystems also play critical cultural roles in BC and their monetary value to tourism is only partially included in these totals (through sport fishing).

Over the past 20 years the wild fishery has declined in terms of both its contribution to the BC GDP and employment, although some individual components are increasing (*e.g.* prawns, Geoduck Clam, Pacific Halibut). Meanwhile aquaculture has nearly tripled its contribution to BC GDP in the same time frame [33]. As a result, published landed values associated with aquaculture are about the same as those from the wild fishery (see Results) and aquaculture now employs slightly more people than does the wild fishery [33].

The wild fishery is for the most part associated with the open coast (outer WCVI and Queen Charlotte Sound, Fig. 1) and is relatively diverse, with no one fishery dominating landed values (see Results). The most important contributors (Pacific Halibut, Geoduck Clams, prawns, crabs, tunas, Sablefish, rockfishes) currently each have landed values in the $20–50 million range [34]. Aquaculture occurs in protected waters: shellfish farming mainly in the northern Strait of Georgia and finfish farms and hatcheries mainly north of that on the northeastern side of Vancouver Island (Fig. 1). In BC, Atlantic Salmon aquaculture clearly dominates all other commercial fisheries (see Results).

Predicting biological impacts due to OA is a highly complex problem that has only become a concern relatively recently (primarily over the past decade). There have been excellent review papers outlining anticipated impacts on a general global scale (*e.g.* [3, 35]) as well as meta-analyses of existing work on the topic (*e.g.* [36]). Cooley and Doney [37] have provided the first estimate of the economic impact of OA, centred on the shellfish fishery, in the United States. However, few studies consider specific ecosystems, particularly in the context of local pH conditions and natural variability, and none focus on the temperate northeast Pacific.

**Fig 3:**
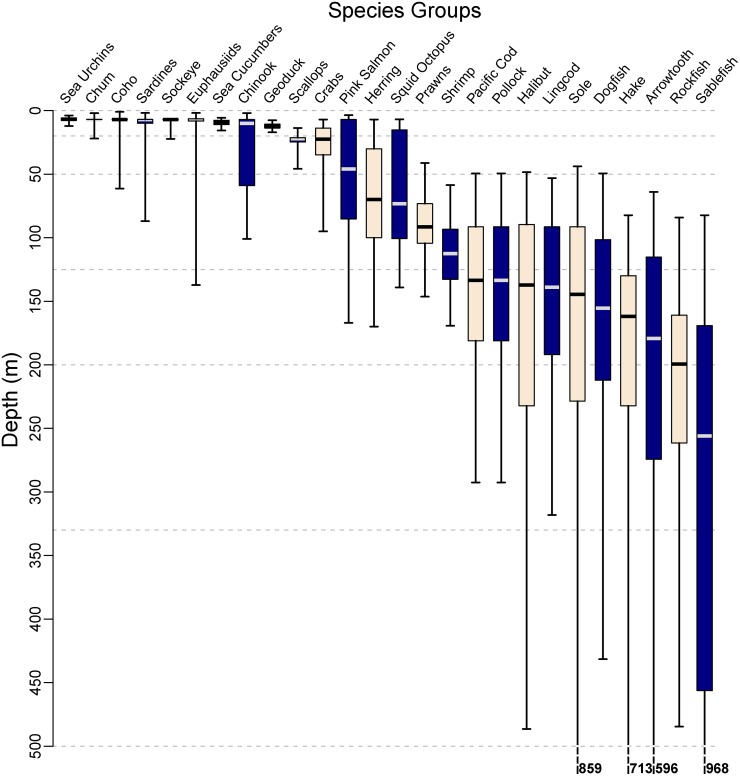
Depth-of-capture, expressed as quantile box plots of depth (m), from fisheries and survey data (where available) for species groups identified in Fig. 4. For each quantile box, the upper whisker, box top, box delimiter (horizontal line), box bottom and lower whisker correspond to the 0.025, 0.25, 0.5, 0.75, and 0.975 quantiles, respectively. Depth quantiles that lie deeper than the figure limit are indicated along the bottom. Horizontal dashed lines correspond to depth zones in Fig. 2. See Methods for data sources. Data and R code for this figure are provided in Supporting Information (S1 Data and S3 Code, respectively).

Here, we examine the potential impact of OA on temperate coastal ecosystems in the northeast Pacific Ocean, with a focus on BC fisheries. To tackle this issue we:

- describe the current marine ecosystem in BC (especially by depth, Fig. 3);
- define the present-day carbon state with depth in local waters (Fig. 2);
- assess the response by marine organisms in this region to OA by investigating existing biological OA impact studies (on local and non-local species) and comparing anticipated changes in acidity (*P*_CO2_) to those currently experienced along the BC coast.

We use the best information available at present to address this problem. The quantitative details, including treatments and measured carbon parameters, of all studies that we used are summarised in S2 Table. We provide specific conclusions ordered by immediacy and relevance to the BC fishery.

## Methods

### Present state of the BC marine ecosystem

**Fig 4:**
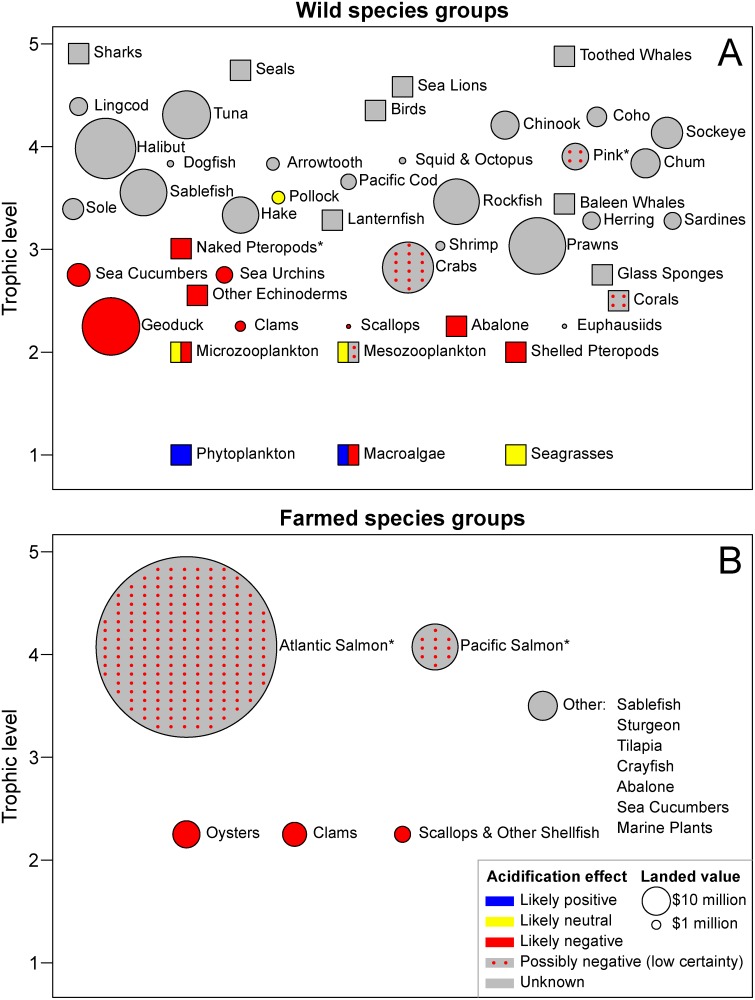
Summary of ocean acidification effects on (A) wild, and (B) farmed species groups in BC waters, including landed value for those that are fished or farmed. Species groups are arranged vertically by trophic level, adapted from output by Preikshot [38] (courtesy of D. Preikshot, Madrone Environmental Services, Duncan BC). Areas of circles are proportional to the landed values in 2011, based on data in [34] (and [39] for euphausiids). Squares represent species groups that are not commercially harvested. Solid colours represent the likely direct effects of ocean acidification (see Results for explanations). Stippling refers to possible effects. For species marked by an asterisk (*), colours represent indirect effects. Data and R code for this figure are provided as Supporting Information (S4 Code).

Marine organisms were assigned to taxonomic groups and sorted by trophic levels adapted from model-derived output for the BC shelf [38] (Fig. 4). We added several taxonomic groups that are commercially fished [34] (*e.g.* sardine, tuna) and unfished (*e.g.* seagrasses, glass sponges) to this list as necessary. To evaluate species abundance and distribution within these groups, we used published literature (both primary and secondary as cited) where available. When literature was not available we consulted Canadian Department of Fisheries and Oceans (DFO) databases and the expertise of individuals active in the field (see Results and *Acknowledgements*). Landed values of fished species were taken from [34] (or [39] for euphausiids).

Species depth distributions (Fig. 3) were obtained from DFO databases (Pacific Biological Station, Nanaimo, Canada). Depths associated with commercially-caught groundfish (compiled by RH, May 1, 2014) and shellfish (compiled by Georg Jorgensen, May 6, 2014) are depths-at-capture, most often a mean of the minimum and maximum depths of fishing events (usually trawl or trap). For the commercial species groups (Fig. 4), depths were selected based on fishing methods specific to each group—Sea Urchins (dive), Euphausiids (nets), Sea Cucumbers (dive), Geoduck Clam (dive), Scallops (dive, trawl), Crabs (trap), Squid & Octopus (dive, trap), Prawns (trap), Shrimp (trawl), Pacific Cod (midwater & bottom trawl), Pollock (midwater & bottom trawl), Halibut (bottom trawl), Lingcod (bottom trawl), Sole (bottom trawl), Dogfish(bottom trawl), Hake (midwater trawl), Arrowtooth (bottom trawl), Rockfish (midwater & bottom trawl), Sablefish (bottom trawl). Depths associated with pelagic species (Herring, Sardines, and Salmon—Chinook, Chum, Coho, Sockeye, Pink) come from two sources: the WCVI Sardine Trawl Survey (spanning the WCVI, Fig. 1: −129.14°W to −124.56°W, 48.32°N to 51.14°N), which occurs mid-summer and is conducted during the night (data compiled by Linnea Flostrand, May 8, 2014), and the La Perouse Survey (spanning the BC coast, Fig. 1: −132.89°W to −123.07°W, 43.58°N to 54.64°N), which is a daytime acoustic trawl survey used to verify acoustic targets (data compiled by Jennifer Boldt, May 14, 2014). The two surveys did not capture any SARA-listed species. Mean depths-of-capture are summarised by quantile boxplots where the box represents 50% of the observations, and the region between the whiskers represents 95% (Fig. 3).

Commercial fishing in Canada is regulated by the *Fishery Act*. Specifically, Section 22 (http://laws-lois.justice.gc.ca/eng/regulations/SOR-93-53/page-6.html) identifies all license conditions that DFO uses to manage gear, monitoring, reporting, harvesting, allocation, and catch requirements. DFO’s Pacific Region Animal Care Committee requires animal-use protocols (Supplementary S1 text), but specifically exempts lethal sampling of fish and invertebrates for stock assessment and sampling from commercial operations where animals are dead or certain to die. Data used here were collected for stock assessments and are therefore exempt from protocols.

### Local inorganic carbon distributions

Published inorganic carbon data (DIC, TA) from the outer BC coast in Queen Charlotte Sound (QCS) [40] and along the WCVI [27, 40] (Fig. 1) are used. These data (174 discrete samples) were collected over the continental shelf, slope and offshore, from the surface to 800 m with greater depth resolution in the top 50 m. The carbonate system was defined from TA and DIC (CO2SYS, [41]) and the constants of [42] with conductivity, temperature, depth and nutrient data that were collected concurrently. These data were sorted into depth intervals defined by local bathymetry relevant to local marine organisms (Fig. 2).

### Responses of marine organisms to OA

We evaluated the potential impact (coded by colour in Fig. 4) of OA on each taxonomic group that occurs in BC, recognizing that uncertainty exists. We also identified the depth distributions that these groups of species occupy, along with associated OA conditions (Fig. 3). Similar to our description of the local marine system, we used published literature where available to assess direct and indirect effects of OA on taxonomic groups. When no publications were available in this rapidly emerging field, we consulted individuals who presented at recent conferences (in particular *2014 Ocean Sciences Meeting*, Honolulu HI and *2014 Salish Sea Ecosystem Conference*, Seattle WA), and we consulted many other experts in their respective fields (cited within Results and *Acknowledgements*).

## Results

There are relatively few published carbon data in BC waters. We use these data [27, 40] to estimate present-day ranges in pH and *P*_CO2_ for depth intervals relevant to local marine organisms (Fig. 2). We then defined three relative *P*_CO2_ levels, which are based on the present-day ranges in Fig. 2, to group the experimental treatments presented in the literature relative to our local waters (Table 1). For example, Pink Salmon (*Oncorhynchus gorbuscha*) generally occupy depths in both the 0–50 m and the 50–125 m zone (Fig. 3) so for these fish present-day *P*_CO2_ in our region is ∼200–1000 *μ*atm (pH ∼7.6–8.4) (Fig. 2) so that a *P*_CO2_ level of 5000 *μ*atm would be the upper limit of an ‘elevated’ (Table 1) treatment.

**Table 1:** Terminology used in the text to quantify levels of *P*_CO2_ used in manipulation experiments. S2 Table provides details for each treatment in each experiment cited.

**Terminology**	***P*_CO2_**
present-day	depends on depth range (Fig. 2)
reduced	0.5× present-day
elevated	2–5× present-day
very elevated	5–10× present-day

Vertical distributions of marine organisms on the BC coast are presented with associated impacts of OA, ordered by trophic level (Fig. 4) in the following sections. Depending on trophic level and group, the amount of information available was variable. For many commercially harvested groups (represented by circles in Fig. 4) excellent data were available (*e.g.* finfish, Fig. 3). On the other hand, abundance and species composition of unfished groups are not well characterised, particularly at lower trophic levels (squares in Fig. 4, *e.g.* microzooplankton, corals). For many organisms important in the region, no published OA related studies exist (grey circles and squares in Fig. 4). Where necessary, we have adopted results from OA studies on species elsewhere that are similar to the ones found locally. These caveats are detailed in each section. Experimental details are summarised in S2 Table.

### Phytoplankton

In the coastal northeast Pacific the predominant class of phytoplankton is diatoms, which are associated with high trophic transfer [43]. Many species (including the dominants: *Skeletonema costatum*, *Thalassiosira* spp., and *Chaetoceros* spp.) occur along the entire coast of BC [44–50]. Large blooms associated with coastal upwelling are often monospecific (*e.g.* [51]), but in our region they appear to be more diverse and occasionally include large numbers of photosynthetic dinoflagellates [46, 52]. Coccolithophorid blooms have been directly observed in more protected regions [50] and by satellite along the entire BC coast during summer [53]; however, coccolithophores (which calcify) are generally assumed to contribute minimally to overall productivity in the coastal zone (roughly landward of the 800 m isobath, Fig. 1) despite their importance further offshore [54]. Primary production by phytoplankton is exceptionally high in the region [27, 47, 55] and ultimately responsible for the high fish yields along our coast [30].

Phytoplankton species that are harmful to higher trophic levels are also common in the region. Large blooms of diatoms from the genus *Pseudo-nitzschia* occur on the outer coast (*e.g.* [56, 57]) while the dinoflagellate *Alexandrium* is more prolific in protected locations [58]. Both *Pseudo-nitzschia* and *Alexandrium* produce neurotoxins that bioaccumulate in higher trophic levels. These toxins can interfere with the reproductive success of fish, seabirds, and mammals and cause mass mortalities [59, 60]. They are also responsible for numerous seasonal shellfish closures in BC (http://www.pac.dfo-mpo.gc.ca/fm-gp/contamination/biotox/index-eng.html). Additionally, significant blooms of *Heterosigma akashiwo* occur in protected waterways [61, 62]. *Heterosigma* releases peroxide free radicals into the water [63], which damage fish gill tissue [64, 65] and cause significant mortality and monetary losses (millions of dollars per year) to salmon aquaculture in BC [66]. Thus, harmful algae already pose a threat to health and food safety along the BC coast [58].

**Direct effects** There have been numerous studies on phytoplankton related to OA (S1 Table) and a variety of responses have been observed depending on the species and the experimental treatment (*e.g.* [67–70]). Although natural conditions in most coastal environments, including the BC coast (Figs. 1 & 2), cover an exceptionally large range in carbon states and consequently pH (*e.g.* [6, 27, 71]), experiments in the field are challenging to complete. Thus, most studies have been conducted in the laboratory, often using a single strain of cultured phytoplankton. Also, because coccolithophores calcify (and at least some are easy to culture), they have been studied disproportionately. We sample a relatively small subset of this body of literature to summarise results of most relevance to the mixed, often diatom-dominated, community in the region and briefly describe the current understanding of the mechanisms involved.

Species specific responses by primary producers, including phytoplankton, to increases in ambient CO_2_ are highly dependent on their carbon-uptake mechanism. Carbon assimilation relies on the enzyme ribulose biphosphate carboxylase-oxygenase (RuBisCO) to fix CO_2_ [72], but this enzyme has a poor affinity for CO_2_ [72, 73]. Over geological times scales (i.e. the last 3.5 billions years), as newer phytoplankton species have evolved, their use of RuBisCO has become more effective [72]. Some have carbon-concentrating mechanisms (CCMs), *e.g.* diatoms [74], to help transport and accumulate CO_2_ to the active RuBisCO site [75]. The most important CCM for phytoplankton involves carbonic anhydrase to convert abundant ${\mathrm{HCO}}_{3}^{-}$ to the limiting CO_2_ [76]. Despite CCMs, many photosynthetic phytoplankters, including some diatoms, appear to be carbon-limited under present-day conditions (*e.g.* [72]).

Because of these limitations in carbon uptake, it is anticipated that OA will increase overall production, which may provide more food to higher trophic levels. However, this increase does not appear to be large. Numerous mesocosm experiments, which use natural assemblages, suggest that regardless of species composition, there may be at most a 10–30% increase in primary production due to OA (*e.g.* [77–80]). In addition, a side-effect of elevated *P*_CO2_ (Table 1) is increased carbon to nitrogen (C:N) ratios in phytoplankton, effectively decreasing its nutritional quality [80].

While it is generally agreed that OA is likely to cause shifts in phytoplankton species composition, it remains unclear what these shifts will be [69]. It is reasonable to expect that species that do not have effective CCMs will do better than species that are already efficient with carbon uptake (diatoms in general). For example, the fish-killing raphidophyte *Heterosigma akashiwo* relies on passive diffusion to obtain CO_2_. As a result it responds strongly (increased rates of growth and primary productivity) to an increase in dissolved CO_2_ [81, 82] regardless of temperature [82]. In contrast, growth rates for some phytoplankton species reach a maximum value at the low end of present-day *P*_CO2_ in the upper mixed layer on the outer BC coast (Fig. 2) assuming a salinity of 31–32 [81]. For other species (including several diatoms) these rates remain invariant under elevated *P*_CO2_ [83].

Competition may be more subtle. For instance, some experiments have shown an increase in the proportion of diatoms relative to smaller phytoplankton with increased *P*_CO2_ (*e.g.* [84]) while others show the opposite effect (*e.g.* [85]). In addition, Tortell *et al.* [86] found that the prymnesiophyte *Phaeocystis* could outcompete diatoms at reduced *P*_CO2_ even though both groups have efficient CCMs. Finally, it has been suggested that at least one motile species (*H. akashiwo*) will swim faster under OA and deepen its vertical distribution [87], which may give it (and any species that can take advantage of its absence nearer to the surface) an additional competitive advantage.

Factors associated with climate change, including OA, are expected to increase the frequency and severity of harmful algal blooms [88]. In addition, the production of potent neurotoxins—domoic acid by common and sometimes prolific diatom species of *Pseudo-nitzschia*, and saxitoxin by dinoflagellate species of *Alexandrium*—has been shown to increase markedly under OA conditions [89–91]. In fact, domoic acid production in (at least some) *Pseudo-nitzschia* spp. increases dramatically (5–50× per cell) as *P*_CO2_ increases [92, 93].

Coccolithophores (prymnesiophytes) are the major calcifiers in the phytoplankton community [94, 95]. The most commonly studied species is *Emiliania huxleyi*, and although it appears to be less prevalent locally in the coastal zone (Fig. 1 in [94]), it plays an important role in the Alaskan Gyre [54]. Numerous experiments (most *in vitro*, some *in situ*) on *Emiliania* have been conducted to determine the effects of carbonate chemistry on calcification. Most (but not all, *e.g.* [96, 97]) suggest decreasing calcification at lower pH values (*e.g.* [70, 98]). Although much remains unknown (*e.g.* [20, 99]), the consensus is that OA will decrease calcification [69]. This observation is reinforced by mesocosm experiments that manipulate coccolithophore populations [67, 100] and by paleolithic records [101].

**Phytoplankton synopsis** We conclude that the overall impact on ecosystems and fisheries due to changes in the phytoplankton community in our region will be negative. While a modest increase in primary production is anticipated (so a direct positive benefit to phytoplankton, Fig. 4A), this increase is not likely to benefit higher trophic levels due to expected shifts in species composition (away from diatoms) and decreased nutritional value of the plankton. More importantly, the fish-killing alga *Heterosigma akashiwo* may gain a competitive advantage, which would seriously threaten salmon aquaculture. In addition, increasing *P*_CO2_ has been shown to alter the mix of neurotoxins produced by genera such as *Pseudo-nitzschia* and *Alexandrium* to favour the more potent forms, posing a significant threat to higher trophic levels and the shellfish industry as well as overall food safety.

### Macroalgae

Three groups of macroalgae are delineated by their pigmentation: green, brown, and red algae, all of which are common in BC. In particular, brown algae constitute the majority of the biomass in intertidal and upper subtidal zones, and are dominated by kelps and rockweeds [102]. Brown algae have soft fleshy morphologies, and both green and red algal groups contain species with hard, calcified structures. Calcified red algae have two morphologies, crust-forming on substrate, and erect and branched. Both red and green algae are found in the intertidal and upper subtidal zones, but red algae extend down to the lower photic zone [103]. The large-blade (brown) macroalgae (*e.g.* *Laminaria*, *Macrocystis*) that form dense kelp forests along temperate coasts, common in BC, are the basis of some of the most productive ecosystems on Earth [103, 104]. These forests provide extensive shelter from predation, desiccation and wave action, as well as food, for hundreds of species with representatives from most taxonomic groups [105]. Calcified red algae provide similar protective structures, that are especially important for invertebrate species (*e.g.* urchins and anemones) [106].

**Direct effects** As with phytoplankton, many macroalgal species use carbon concentrating mechanisms (CCMs) to help transport and accumulate the CO_2_ required for carbon assimilation [107]. Those relatively rare species without CCMs (most of which are red algae) rely on passive diffusion of CO_2_ [108, 109] and so may experience enhanced photosynthesis and growth under OA, whereas those that have CCMs are likely to show no, or only small, positive effects due to reduced energy expenditure [107, 110]. Responses to elevated *P*_CO2_ (Table 1) may be more significant at depths where light levels are reduced because energy constrains photosynthesis and CCMs are energetically expensive, though these effects are likely to be species-specific [110]. In addition, UVB (Ultraviolet B, 280–315 nm) exposure near the water surface tends to be harmful to some macroalgae, reducing the positive response to elevated *P*_CO2_ [111]. The ultimate effects of OA on photosynthesis and growth of macroalgae will likely depend on interactions with light exposure, UV radiation, and other stressors. There has been less research concerning reproduction and life stages; however, it has been suggested that OA will result in reduced gametophyte growth of giant kelp [112].

For calcifying macroalgae, elevated *P*_CO2_ affects the ability to build and maintain the calcified component of their tissues [108]. For example, Hofmann *et al.* [113] observed reduced calcification and growth for a cosmopolitan species of red algae when exposed to elevated *P*_CO2_ over a 4-week period (S2 Table). Calcifying red algae are particularly sensitive to OA because unlike most calcifying green algae and invertebrates, red algae deposit a high-magnesium form of calcite into their cell walls, that is more soluble in acidified water than other forms of calcite [28]. However, Kroeker *et al.* [36] found no consistent change in calcification at elevated *P*_CO2_ levels for a suite of calcifying macroalgae, perhaps because many species are able to generate microenvironments suitable for calcification despite increases in ambient *P*_CO2_ [114–117]. Indeed, the observed reductions in growth with elevated *P*_CO2_ (*e.g.* [113]) may result from the increased dissolution of carbonate skeletons rather than reduced production [117]. These effects are likely to interact with other stressors, such as UV radiation and temperature [118]. For example, Gao and Zheng [119] suggest that the carbonate skeleton of the same red algal species serves as a protective layer against UV; thus, CO_2_ induced shell dissolution may increase vulnerability to detrimental effects of UV radiation [119].

**Indirect effects** Changes in macroalgal community composition are anticipated given the diversity of responses to OA among species. In general, non-calcifying macroalgae (especially those that rely on diffusion of CO_2_ instead of CCMs) are expected to experience increased competitive success compared with calcifying macroalgae [110], resulting in an overall shift of community composition toward non-calcifying species [36]. Furthermore, studies on CO_2_-enriched waters surrounding seafloor vents elsewhere support this hypothesis [120]. Most research has focused on losses of crust-forming calcified red algae in particular and replacement with non-calcifying turf-forming algal communities (*i.e.* species that reach heights of <15cm [121]) [36, 115, 122]. In BC, crust-forming red algae release chemical cues that play an important role in the settlement of some invertebrate larvae (*e.g.* abalone [123, 124]), and they bond substrata to provide stable habitats for other benthic species [106], but the resulting ecosystem effects under OA remain highly uncertain. Likewise, the ecological effects of possible declines in erect calcified red macroalgae and replacement by fleshy macroalgal species have received little attention (but see [113, 125]).

In addition to competition, herbivory is another key factor structuring macroalgal communities [126]. Rates of herbivory on macroalgae depend on palatability and the presence of hard carbonate structures for algal defence [127]. OA may reduce structural protection thereby increasing grazing on calcified species [115]. For non-calcified species, OA may increase C:N ratios possibly reducing palatability and hence grazing pressure [115]. However, OA will likely be detrimental to many herbivores, especially calcified species such as echinoderms and molluscs (see below), with resulting beneficial effects on some macroalgal species (*e.g.* [128], Mediterranean Sea, S3 Table).

Given these potential impacts, Harley *et al.* [115] suggest that in the California Current ecosystem, which includes the WCVI, OA may result in a shift from diverse nearshore communities consisting of kelp canopies, understory turf assemblages, crust-forming calcifying algae, and calcifying invertebrates (*e.g.* urchins), to communities dominated by kelp and macroalgal turfs. Where kelp canopies have been lost due to other natural or anthropogenic disturbances (*e.g.* indirect effects of commercial harvest of fish species as found for large regions of the northeast Pacific, [129]), OA may prevent kelp recovery by facilitating expansion of algal turfs which inhibit kelp recruitment [130], as found along the Australian coast [131]. Kelp is the dominant primary producer among macroalgal species in BC, providing food and habitat for commercially important fish species, such as Pacific salmon [132, 133]. However, because responses of benthic communities to OA are highly species-dependent, the results of these studies cannot be extrapolated to other regions with high confidence [115].

In addition to community-level effects from altered competition and herbivory, OA may slow decay rates of some kelp species including those commonly found in BC (*e.g.* bull kelp, *Nereocystis leutkeana*), which could indirectly affect detritivore consumption and nutrient cycling [111]. This delay may result in the accumulation of phytodetritus, possibly reducing food availability for consumers in nearshore waters.

**Macroalgae synopsis** The direct effect of OA is hypothesised to be positive on non-calcifying species due to enhanced availability of CO_2_ for carbon assimilation, but negative for calcifying species due to reduced growth and dissolution of protective shells (Fig. 4A). Community composition may shift from calcifying macroalgae species toward non-calcifying species, with an inhibition in the recovery of depleted kelp populations. However, community-level responses will depend on the extent of grazing on fleshy, non-calcifying species, possible changes in grazing due to OA-impacts on invertebrate herbivores, and the expansion of algal turfs. Responses of benthic communities to OA are highly species-dependent, limiting confidence in generalisations and extrapolations among regions and studies.

### Seagrasses

Seagrasses belong to a small group of marine angiosperms comprising 60 species worldwide [134]. In BC, there are only two species of eelgrass—the native *Zostera marina* and the introduced species *Z. japonica*—and three species of surfgrass all belonging to the genus *Phyllospadix* [135]. Seagrass beds are well-known as nurseries for juvenile fish and invertebrates [136]. Another advantage conferred by seagrass beds is their ability to modify the seawater carbonate system, increasing aragonite saturation states within their confines [137], which might offer calcifying organisms refugia from the effects of OA.

In contrast to most macroalgae, seagrass cannot take advantage of the abundant ${\mathrm{HCO}}_{3}^{-}$ [138] and so increase their photosynthetic rate when DIC becomes more abundant [139]. With more DIC, seagrass are better able to compensate for light attenuation [139]. As a result, increased *P*_CO2_ may foster the growth of seagrass beds, despite worldwide losses of seagrass ecosystems due to anthropogenic disturbances along coastal environments [134]. However, OA-related reductions in phenolic compounds [140], which protect seagrasses against herbivory, may result in increased grazing pressure under increased *P*_CO2_. The evidence for decreasing phenolics in seagrass under OA is limited and contrary to the trend of increasing phenolics in terrestrial angiosperms under increased atmospheric CO_2_ [140].

**Seagrass synopsis** Seagrasses will likely benefit from increased *P*_CO2_ because higher DIC helps them compensate for light limitation; however, a decrease in protective phenolic compounds may offset any benefit due to increased grazing. The net effect of increased OA will likely be neutral for seagrasses.

### Microzooplankton

Microzooplankton (20–200 *μ*m) include heterotrophic protists such as ciliates and non-photosynthetic dinoflagellates. Typical ciliate genera along the BC coast include *Strombidium*, *Tintinnopsis* and *Strobilidium* [141] while the heterotrophic dinoflagellate species belong chiefly to *Protoperidinium*, which feeds almost exclusively on diatoms [142], and *Gyrodinium*. In nearshore waters, microzooplankton can be very abundant, depending on the time of year and food source (*e.g.* [44]). More importantly, fluctuations in microzooplankton populations, tightly coupled to phytoplankton, can have a large effect on pelagic ecosystems [143] and can influence the success or failure of fish recruitment [144].

**Direct effects** There are no studies that test the direct effects of OA on individual microzooplankton species. That said, foraminifera are amoeboid protists that form CaCO_3_ shells and, like coccolithophores, are probably at risk from OA (*e.g.* [145]). There is also speculation that microzooplankton motility might be affected by OA [146], with the closest evidence coming from the study of the photosynthetic flagellate *Heterosigma* that demonstrated an increase in swimming speed and an increase in downward migration [87]. Large-scale mesocosm manipulations and on-board experiments that compare present-day and elevated *P*_CO2_ (Table 1) have found conflicting results—(i) no shifts in composition or abundance [147–149], (ii) almost identical succession patterns [150], and (iii) significant increases in heterotrophic dinoflagellate abundance [151, 152], although in the former (i.e. [151]) an increase in the prey species of diatoms was likely responsible.

**Microzooplankton synopsis** Based on the limited studies for microzooplankton, we expect that most species will be unaffected by OA, except through changes to their prey (phytoplankton). Direct OA effects will likely have a negative effect on foraminifera through reductions in CaCO_3_ shells.

### Mesozooplankton

In our region, the zooplankton community is strongly dominated by calanoid copepods [153, 154]. Important species include *Neocalanus plumchrus*, *Acartia longiremis* and *Pseudocalanus* spp. [153, 154]. In protected regions like the Strait of Georgia (Fig. 1) *Calanus pacificus* is also important [154], while on the outer shelf *Calanus marshalae* is significant [153]. Some species spend part of their life cycles (that includes egg production) in relatively deep waters, >300–500 m (*e.g.* *Neocalanus plumchrus* and *Calanus pacificus*) while others, like *Acartia longiremis*, are always found above ∼50 m. Zooplankton productivity is variable and appears to be changing over time [153], with species composition dependent on temperature [154]. Mesozooplankton provide the main trophic link connecting phytoplankton and microzooplankton with larger oceanic predators [155]. They are critical for several commercially-valuable fish species that prey on them directly, such as Pacific Herring, Pacific Hake, Pacific Sardine, various salmon species, and Spiny Dogfish (*Squalus acanthias*) [155].

**Direct effects** Only *Calanus pacificus* has been studied locally so we include experiments on copepods found elsewhere from the common genera *Acartia* and *Calanus*. Although responses to acidic conditions can be species-specific, even within genera (*e.g.* [156]), our summary provides a general indication of possible effects on the mesozooplankton community in our region.

Most OA related mesozooplankton research involves eggs and/or survival rates within individual stages. Egg production rates of adult females appear unaffected by increased *P*_CO2_ (even under very elevated conditions, Table 1) [156–160], although *P*_CO2_-induced increases or decreases were observed depending on temperature [161]. On the other hand, egg hatching rates may decrease with OA [156–160], although increases have also been observed [161]. However, it is possible that hatching is simply *delayed* and so not observed in short-term experiments [160]. Effects of OA on overall egg hatching success are uncertain. In Puget Sound, WA (Fig. 1), egg hatching in *Calanus pacificus* is reduced under elevated *P*_CO2_ (Anna McLaskey, pers. comm., University of Alaska, Fairbanks AK), whereas egg hatching success in *Calanus helgolandicus* (found in the North Atlantic) appears unaffected [162]. For copepod embryos, survival rates appear unaffected by OA, while developmental rates may decline [163]. In adult copepods, survival rates are not significantly affected even under very elevated experimental conditions (except for one species) [156, 157, 159].

Although impacts on individual life stages may not be significantly different from a control scenario, the cumulative impacts may be significant. In addition, the studies thus far have been relatively short-term, and do not consider the possibility for copepods to respond to environmental changes through adaptive evolution [161]. The lack of detailed information on potential effects on zooplankton physiology “currently restricts our ability to reliably predict future impacts” [162].

**Mesozooplankton synopsis** For copepod species from the genera *Acartia* and *Calanus*, adult survival rates and egg productions rates appear unaffected by OA, even when *P*_CO2_ is ‘very elevated’ (Table 1), whereas egg hatching rates are negatively affected and egg hatching success remains uncertain. Cumulative impacts across life stages are unknown. Thus, the effects of OA on mesozooplankton will likely be neutral and possibly negative (Fig. 4A).

### Pteropods

In BC waters only three species of pelagic snail, or pteropod, have been regularly observed [164]. *Limacina helicina* (shelled) is by far the most common of these three, occurring throughout most of the year, generally in the upper 100 m [164] and occasionally forming strong blooms (> 1000 m^−3^) which can dominate the plankton (M. Galbraith, pers. comm., Institute of Ocean Sciences, Sidney BC). *Clione* spp. (naked) is also often present, although at significantly lower numbers. These two species are common in the Strait of Georgia and less so in Hecate Strait (Fig. 1); they are also found on the outer BC shelf and in the Alaskan Gyre (M. Galbraith, pers. comm.). *Clio pyramidata* (shelled), a subtropical species, is present only episodically along the WCVI [165]. Pteropods are an important food source for fish (especially juvenile salmon [166]), birds and marine mammals [167, 168]. Most pteropods produce aragonitic shells [167] and those that don’t (naked pteropods) feed almost exclusively on the shelled species, making all pteropods susceptible to OA [164].

**Direct effects** It is difficult to keep pteropods in laboratory conditions [164] due to their delicate feeding structure [167]. Thus, few controlled experiments on live animals have been made until recently, and sample size remains limited. Most of these experiments have been conducted on (variants of) *L. helicina* harvested from Arctic and Antarctic waters (S2 Table).

Shells of dead pteropods dissolve in waters undersaturated with respect to aragonite, (*e.g.* [169, 170]) as expected. Live individuals, which may form protective biological coatings on the exterior of their shell [171] and/or actively counteract dissolution [170] also show evidence of dissolution when harvested from waters under, or near, saturation with respect to aragonite [172–174] (S3 Table). Similarly, live individuals incubated for short periods under the high end of present-day *P*_CO2_ (0–100 m, Fig. 2) and elevated *P*_CO2_ (Table 1) show reduced calcification (*e.g.* [170, 175]; S2 Table). In one experiment the larval state failed to calcify at all [176].

Despite the negative impacts on shell quality and maintenance, many (and in some cases all, *e.g.* [175]) animals studied survived their respective treatments (*e.g.* [170, 177]). However, the reduction of shell formation will impact the pteropods’ ability to control buoyancy and withstand predation [167]. In addition, as *P*_CO2_ rises, increased energetic costs associated with maintaining their shells are likely, particularly as temperature increases [170]. The ability to supply energy to perform these (and other) tasks may be suppressed, [178] although some pteropods are likely to be more resilient than others (*e.g.* [179], S2 Table).

**Pteropod synopsis** In summary, there is a clear cause for concern about the future of pteropods and the animals that depend on them. Although in the last several decades pteropods make up, on average, only about 5% of the average annual zooplankton biomass in BC waters (M. Galbraith, pers. comm.), they are an important food source for juvenile Pink Salmon [166] and are related to Pink Salmon survival [180] (see Fish—Indirect effects). Already in our region, where aragonite saturation horizons are frequently shallower than 100 m [11, 31, 32], numbers of the most common pteropod have declined significantly [164].

### Molluscs

Molluscs comprise a diverse group of organisms that includes a variety of shellfish as well as predators such as squid and octopus (and pteropods, above). In the northeast Pacific, mussels dominate rocky intertidal zones (*e.g. Mytilus californianus* [181]) while oysters (mainly the Pacific Oyster, *Crassostrea gigas*), clams (family Veneridae) and cockles (family Cardiidae) are commonly found on beaches [182]. Geoduck Clams and scallops live significantly deeper (∼10–20 m and 15–45 m, respectively) as do squids and octopuses (∼15–140 m, Fig. 3). Shellfish consume plankton through filter-feeding and are able to significantly reduce plankton concentrations on a local scale (*e.g.* [183]), making them strong indicators of water quality [184, 185]. In turn, shellfish are preyed upon by many animals including sea otters, octopuses, seabirds and sea stars [186, 187].

The annual landed value of molluscs harvested from wild and farmed fisheries in BC is $63 million (Fig. 4), of which 66% is Geoduck Clam (*Panopea abrupta*). Other major harvested clams are Manila Clam (*Venerupis philippinarum*), Native Littleneck Clam (*Leukoma staminea*), Butter Clam (*Saxidomus gigantea*) and Varnish (Savoury) Clam (*Nutallia obscurata*) [188]. The Pacific Oyster was introduced into BC waters in the early 1900s and is used in aquaculture, while the native Olympia Oyster (*Ostrea conchaphila*) is no longer harvested [189, 190] and is listed as *Special Concern* under the Canadian Species at Risk Act (SARA). There are small fisheries for Pink Scallop (*Chlamys rubida*) and Spiny Scallop (*Chlamys hastata*) [191]; a commercially-developed hybrid called “Pacific Scallop” (*Patinopecten caurinus x yessoensis*) is used in aquaculture. There is a small but growing mussel industry, no harvest for Northern Abalone (*Haliotis kamtschatkana*) as it is listed by SARA as *Endangered*, and minor harvests for squid and octopus.

**Direct effects** Shelled molluscs calcify internally and actively increase pH at that site to do so, making them directly vulnerable to OA [16, 17]. Larval shells are particularly vulnerable since they are mostly composed of aragonite [192, 193] and for at least a few species the initial deposit is amorphous CaCO_3_ (the least stable form of CaCO_3_) [192]. By adulthood, shells are composed of aragonite and/or calcite, depending on the species [192, 193]; *e.g.*, oyster shells are mainly calcite [194]. To deal with vulnerability at the larval stage (*e.g.* [195]), mollusc aquaculture in the northeast Pacific relies on hatcheries (often with controlled conditions) to rear larvae that are then distributed to growers.

Experiments to quantify OA effects on shellfish have yielded a range of conclusions [36, 196]; however, with the advancement of the field, results are beginning to converge. Kroeker et al. [36] found that OA significantly reduced calcification (by 40%), growth (by 17%) and development (by 25%) in molluscs. Another recent review [197] found that 37 of 41 studies on calcification by molluscs reported significant negative effects following exposure to increased CO_2_ levels. Here we summarise experiments performed on species that are found in the northeast Pacific and elsewhere (*e.g.* scallops). There have been no studies on Geoduck Clams (despite their commercial importance), or on BC scallop species.

Experiments on fertilisation in Pacific Oyster have produced mixed results. Both sperm swimming speed and egg fertilisation success can be unaffected [198] or decline [199, 200] under elevated *P*_CO2_ (Table 1). Within two days of fertilisation, Pacific Oyster larvae precipitate >90% of their body weight as CaCO_3_, using limited energy reserves in eggs [17]. Early development (up to 8 h) remains unaffected at elevated *P*_CO2_ [201]; however, the number of embryos reaching the planktonic ‘D-veliger’ larval stage declines [199–201]. Elevated *P*_CO2_ increases the number of larvae with shells one day after fertilisation (due to an enhanced metabolic rate), yet decreases it three days after [202]. Larval survival of Pacific Oysters is unaffected by *P*_CO2_ after three and 16 days [202, 203]. Species that do exhibit a decline in larval survival are Northern Abalone [204] and Bay Scallop (*Argopecten irradians*) [205].

Metamorphosis from larvae to juveniles is affected differently for different species under elevated *P*_CO2_. For Olympia Oyster, the proportion of metamorphosing larvae declines [206, 207] and size at metamorphosis decreases [206]. Similar results, plus a delay in metamorphosis and reduction in survival, are usually seen for Bay Scallop [205, 208–210]. However, for Northern Abalone from the WCVI the proportion of metamorphosing larvae is unaffected [204]. Increased abnormalities in larvae have been observed under elevated *P*_CO2_ in Pacific Oyster [199–201] and Northern Abalone [204]. In the latter species, shell abnormalities increased substantially, occurring in 99% of larvae at *P*_CO2_ 1800 *μ*atm [204]. These abnormalities did not appear to affect survival rates in the laboratory, but in the field the abnormal larvae would be more susceptible to predation [204].

The size of D-veliger larvae of Pacific Oyster decreases [199–202, 211] and shell growth of later larval stages generally declines [199, 201] under elevated *P*_CO2_, though not always [199, 203]. Decreases in larvae shell growth also occur in Olympia Oyster [207, 212], Northern Abalone [204] and Bay Scallop [208–210]. Molecular analyses show that expression of proteins related to calcification and cytoskeleton production can be severely suppressed under high *P*_CO2_ [211]. For Northern Abalone larvae, settlement (attachment to the experimental container) is unaffected by *P*_CO2_ [204]. Additional effects on other larvae include decreased O_2_ consumption and feeding rates [203], and reduced lipid content [209, 210].

Shell growth and calcification of juvenile and adult molluscs under OA remains uncertain due to limited studies with contrasting results. Pacific Oyster juveniles exhibit increased expansion of shell area (but not thickness) under reduced pH, despite declines in O_2_ consumption and feeding rates of larvae [203]. In juvenile Bay Scallops, elevated *P*_CO2_ (Table 1) does not affect shell and tissue growth but does reduce survival [209]. Declines in calcification rates have been observed for Pacific Oyster juveniles and adults under elevated *P*_CO2_ [213] and for adult Zhikong Scallops (*Chlamys farreri*) under reduced pH [214].

The byssal threads that mussels use to attach themselves to rocks or vertical lines in aquaculture must be robust so that they do not drop off or get ripped off. The threads of the common mussel (*Mytilus trossulus*) have been shown to weaken under elevated *P*_CO2_ [215], although they may be more sensitive to temperature during during short-term fluctuations typical of local inlets (L. Newcomb, University of Washington, Seattle pers. comm.).

Metabolic rates of juveniles and adults appear to be generally unaffected by OA alone [216–218]. Also unaffected, at least in juvenile King Scallops, are clearance rates, growth rates, the ratio RNA:DNA (suggesting no effect on growth potential) [217], and various measures related to ‘clapping’ (rapid closing used for locomotion) by adults—frequency, recovery time between claps and clapping fatigue [218]. The latter study, however, did find a reduction in the force exerted by the clapping under elevated *P*_CO2_, which could reduce the scallops’ ability to escape predators.

As above, the larval stage is vulnerable to OA. South of BC, at a hatchery for Pacific Oyster in Oregon (USA), carbonate levels experience large fluctuations due to strong coastal upwelling [195]. Negative correlations were found between the aragonite saturation state (Ω_arag_ of water in which larvae were spawned and reared, and the resulting larval production and mid-stage growth [195]. In the laboratory, the shell growth rate of juvenile Olympia Oysters depends on pH exposure at the larval stage but not at the juvenile stage [212]. To test such carry-over effects in a natural system, Olympia Oyster larvae were reared under different *P*_CO2_ levels, then transferred to field sites after metamorphosis [206]. Juvenile survival was not significantly different between the two larval treatments, but the elevated-*P*_CO2_ larvae yielded smaller juveniles, suggesting that they suffer irreversible damage (*e.g.* energy deficit, abnormality, inability for compensatory growth) [206].

**Indirect effects** Changes in species composition can be expected under OA. Few studies explore these changes for molluscs, however it has been shown that Eastern Oyster larvae (*Crassostrea virginica*) have higher survival rates than Bay Scallops under elevated *P*_CO2_, which is the opposite of the present-day *P*_CO2_ result (and in the absence of brown tides—in this study caused by a temperate phytoplankton species not found in the northeast Pacific) [210]. Thus, scallops may be affected by OA more than oysters. Scallops are also sensitive to other anthropogenic stressors, such as eutrophication [219], while the impact of these conditions on oysters and other shellfish was not investigated.

OA may increase the vulnerability of shelled molluscs to predation by thinning their protective shells and may also cause food web shifts. For example, Boring Sponges (*Cliona celata*) can bore twice the number of holes in Bay Scallop shells, and remove twice the weight of shell, at pH 7.8 compared to pH 8.1, despite taking longer to attach themselves to the shells [220]. Negative impacts on molluscs could also have large unintended consequences for other species [221]. Shell production and aggregation provide refuge for other organisms such as sponges and crabs, and introduce complexity and heterogeneity into benthic environments, with heterogeneity being important for maintaining species richness [221]. Thus, the direct effects of OA on molluscs may have detrimental effects at the ecosystem level.

**Squid and octopus** In BC, there are at least 30 species of squid and eight species of octopus [222], none of which have been studied for OA effects. Common species in BC waters are Opal Squid (*Loligo opalescens*) and Northern Giant Pacific Octopus (*Enteroctopus dofleini*). Similar to the otoliths of fish (see below), squids have internal calcified structures called statoliths used for sensing gravity and movement [223]. Under elevated *P*_CO2_ statoliths in embryos of the European Squid, *Loligo vulgaris*, are significantly larger than those formed under present-day *P*_CO2_ [224]. At higher *P*_CO2_ (still in the elevated range—Table 1), Kaplan *et al.* [225] observed reduced surface area, malformation, and abnormal crystalline structure in statoliths of Atlantic Longfin Squid, *Doryteuthis pealeii*. Aside from calcification, elevated *P*_CO2_ also leads to increased heavy metal retention in the protective eggshells and changes to the bioaccumulation of silver, mercury and cobalt in larval tissue [224]. Additionally, elevated *P*_CO2_ depresses metabolic rates in pelagic squids (*e.g.* [226]). The ultimate effect on fitness is not known.

**Mollusc synopsis** We conclude that the effects of OA on shelled molluscs will be negative based on available studies on oysters, scallops, abalone and mussels (Fig. 4). These negative effects occur at various life-history stages, and go beyond direct effects on calcification of larvae, *e.g.* reduced oxygen consumption and feeding rates of larvae and delayed behavioural responses of adults. It is generally anticipated that effects on larval survival rate and reproduction rate will directly influence population size, population distribution and community structure [227]. No experiments were found on local clam species (including geoducks) but given the results on other molluscs [36] we anticipate that they will also be negatively affected by OA, while effects on squid and octopus remain uncertain (Fig. 4).

### Sponges and Coldwater Corals

Sponge reefs are globally unique to the northeast Pacific coast [228–230] and all four groups of cold-water corals: octocorals, stylasterids, stony and black corals, are present in the region. They occur where productivity and water flow are high (*e.g.* they are especially dense on seamounts and the heads of canyons, Fig. 1) and from the surface to depths >2000 m [231]. However, due in part to the depth range, very few benthic habitat mapping data exist along the BC coast (*e.g.* [232], Kim Conway, pers. comm., Pacific Geoscience Centre, Sidney, BC) and so we have used these data and the expertise of others to provide our own general description (below). Sponges and cold-water corals form important habitat for many marine organisms including species of fish that are commercially important (*e.g.* the rockfish Pacific Ocean Perch) in our region [233–236].

The coral and sponge contribution to the benthic fauna in BC appears to be patchy but diverse, based on: DFO trawl survey and observer records [237], comparison with neighbouring regions (*e.g.* [238, 239]), isolated studies (*e.g.* [229, 235]), anecdotal evidence (Lynne Yamanaka, pers. comm., Pacific Biological Station, Nanaimo, BC), and modelling work (*e.g.* [240]). This collection is likely dominated by siliceous sponges, and isolated stands of flexible corals with partly organic skeletons (octocorals), more specifically members of the diverse group Alcyonacea (*e.g.* large tree form coral) and pennatulaceans (sea pens and whips). Alcyonacea and solitary glass sponges occur on bedrock, mainly deeper than ∼200 m, while pennatulaceans and glass sponge reefs grow on flat sediment, generally shallower than ∼200 m [241].

Stylasterids (*e.g.* [242]) and stony corals (Scleractinia) also occur [237, 243], but primarily in small, solitary patches. The reef-forming scleractinian *Lophelia pertusa* has been found [244], but is rare, possibly influenced by the already low aragonite saturation states in this region [245]. Black corals, which do not calcify and are made of organic proteins, are also present below 500 m [237].

**Direct effects** OA studies have focused on stony corals, primarily *Lophelia pertusa*, which is entirely aragonitic. They show an increased energetic cost for calcification in *L. pertusa* with decreasing pH (and Ω_arag_ [246, 247] (S2 Table); however, *L. pertusa* may adapt to moderate decreases in pH given sufficient time [248] (S2 Table). The holdfasts and some parts of the structure of many octocorals are also made of aragonite [249]. Similarly, some stylasterids precipitate aragonite as well as calcite [250]. However, neither octocorals nor stylasterids have been studied with respect to OA to date. Likewise, there are no OA studies specific to glass sponges.

**Sponge and coral synopsis** The OA response of the cold-water corals most common in our region (octocorals) has not yet been studied. While the skeletons of these corals are partly organic, they also calcify and so may be affected by OA at some level (Fig. 4A). There are no OA studies on glass sponges to date. Loss of coral and sponge habitat would have a negative impact on many fish species, particularly juvenile rockfish [233–235].

### Echinoderms

Echinoderms form a marine set of invertebrate animals with ∼7000 known species worldwide [251] and 217 species recorded in BC [252], half of which occur exclusively at depths > 200 m [253]. The echinoderms comprise five classes: (i) echinoids (sea urchins and sand dollars), (ii) asteroids (sea stars), (iii) holothuroids (sea cucumbers), (iv) crinoids (sea lilies and feather stars), and (v) ophiuroids (brittle stars). A few are considered to be “keystone” species, such as the Purple Sea Star (*Pisaster ochraceus*) [254, 255], which is common along the BC coast. Echinoderms modify ecosystems (*e.g.* by mixing and transforming sediments, grazing kelp forests, preying on mussel beds) and provide food for carnivorous fish, shellfish, and marine mammals (*e.g.* sea otters prey heavily on sea urchins and sea cucumbers). In addition, sea stars and sea urchins act as important grazers in the sub-littoral zone [256].

**Direct effects** Green and Red Sea Urchins (*Strongylocentrotus droebachiensis* and *S. franciscanus*, respectively) harvested in BC generate significant income (Fig. 4A). Clark *et al.* [257] found that larval growth and skeletal calcification were reduced at lower pH levels for select species (see S2 Table) ranging from the tropics to the poles; no changes in skeletal morphology occurred. Studies on shell thickness are confounded by effects of diet and experiment length [125, 258], but urchins have higher growth rates when fed on calcifying algae and may derive some portion of essential elements (*e.g.* calcium, magnesium) from the algae [258]. Therefore, sea urchins may suffer as the proportion of calcifying macroalgae in their diet declines due to direct OA effects on these algae (see Macroalgae section above). In long-term studies, sea urchins have shown an ability to adapt to elevated *P*_CO2_ (Table 1); however, in the transition to new OA conditions, species may suffer from life-cycle carry-over effects. For instance, Dupont *et al.* [259] demonstrated that under elevated *P*_CO2_ females acclimated for four months experienced a 4.5 decrease in fecundity and produced offspring that suffered 95% juvenile mortality; however, these effects disappeared after acclimitisation for 16 months (S2 Table). OA may also influence reproduction in echinoderms. For example, as *P*_CO2_ increases under OA, higher sperm concentrations are necessary to achieve high fertilisation success in the sea urchin *S. franciscanus*, and the egg’s mechanism for blocking fertilisation by multiple sperm cells becomes slower [260].

A number of studies have used genetic markers to infer the possible physiological effects of OA in sea urchins (see S2 Table). O’Donnell *et al.* [261] measured the change in expression of a molecular helper-protein in *S. franciscanus* and suggested that the ability to handle temperature stress would be reduced under OA. Todgham and Hofmann [262] measured changes in ∼1000 genes of the sea star *S. purpuratus* and found reduced expression under elevated *P*_CO2_ in four categories—biomineralisation, cellular stress response, metabolism, and apoptosis (cell death). Also for this species, elevated *P*_CO2_ triggered changes in 40 functional classes of proteins, affecting biomineralisation, lipid metabolism, and ion homeostasis [263].

Giant Red Sea Cucumber (*Parastichopus californicus*) harvest also provides significant income in BC (sea cucumbers, Fig. 4A) but there are no studies on OA effects for this species. Elsewhere, a single study found that sperm motility of a reef-dwelling sea cucumber species (*Holothuria* sp.) was impaired at pH values <7.7 [264]. Elevated *P*_CO2_ and temperatures have been shown to have positive and additive effects on the relative growth of the keystone sea star *Pisaster ochraceus* [265]. Under increased *P*_CO2_, calcification is reduced [265]; however, growth rate remains unchanged as the endoskeleton is primarily composed of soft tissue with relatively small calcareous elements for rigidity and protection. Brittle stars (ophiuroids) are commonly found in the region, but the effects of OA have only been studied in species found elsewhere. In the eastern Atlantic Ocean, keystone brittle star *Ophiothrix fragilis* was found to be especially sensitive to small changes in pH [266], with 100% mortality of larvae at pH 7.9 vs. 30% mortality in the control (pH = 8.1). Finally, while Dupont *et al.* [251] found that echinoderms studied to date are relatively robust to OA effects, they conclude that the overall impact of OA on this group will be negative and suggest that associated ecosystem impacts may be more severe.

**Indirect effects** Declines in some echinoderms may affect the predators that depend on them, but ecosystem effects remain unknown. For example, on our coast, various nearshore rockfish and numerous flatfish prey on ophiuroids [267], although they only form an important component of the diet for China Rockfish (*Sebastes nebulosus*), Flathead Sole (*Hippoglossoides elassodon*), and Southern Rock Sole (*Lepidopsetta bilineatus*) [267]. Additionally, the deep-water rockfish Longspine Thornyhead (*Sebastolobus altivelis*) relies on brittle stars for a large proportion of its food [268]. In the eastern Atlantic, the inevitable decline in pH may lead to the disappearance of the keystone brittle star *O. fragilis*; the impact on the ecosystem is not really known [266].

**Echinoderm synopsis** Although many echinoderms have not been studied, the existing evidence indicates significant negative effects due to OA, especially at early life stages. Thus, we suggest that this group will be affected negatively (Fig. 4A). Of more concern are the anticipated negative impacts on ecosystems, *e.g.* declines in the population of a keystone species like the Purple Sea Star would have wide-ranging effects on the food web.

### Crustaceans

Marine crustaceans are represented in BC by copepods [269], krill (euphausiids) [39], barnacles [270], shrimps, prawns and crabs [271]. Copepods (see Mesozooplankton) and krill form a substantial biomass in the oceans and provide an important source of food for upper trophic levels in temperate marine foodwebs and act as important grazers (e.g., [272]). Crabs are found in the upper 50 m, while adult prawns (*Pandalus platyceros*) and adult shrimp (mainly Smooth Pink—*Pandalus jordani* and Sidestripe—*Pandalopsis dispar*) are deeper (∼100 m and 120 m, respectively: Fig. 3). Krill, primarily *Euphausia pacifica*, perform strong diel vertical migration from the surface to depths exceeding 100 m. Krill is harvested on a limited basis in the Strait of Georgia and various inlets [39]. Prawns and shrimps, which are farmed extensively in other parts of the world, are only harvested from the wild in BC; the prawn fishery is substantial (∼$40 million, Fig. 4) [34]. The crab fishery in BC is also valuable (∼$33 million) [34, 273], with Dungeness Crab (*Cancer magister*) being the most important commercial species.

**Direct effects** Crustacean exoskeletons, composed of chitin and CaCO_3_ [274], are generally considered to be unaffected by OA. In fact, evidence suggests that this protective covering actually serves as a buffer to the corrosive nature of OA, and some crustaceans can use the increased DIC in seawater to fortify their shells through calcification [275]. This enhancement of the shell contrasts with shell dissolution in molluscs (see Molluscs Section), and is likely due to some crustaceans (crabs, lobsters) having an efficient proton-regulating mechanism [275]. Despite the advantage of localised pH-regulation, the calcification response appears to depend on a variety of additional factors: external organic coatings, skeletal mineralisation composition (*e.g.* magnesium content in calcite), and the degree to which amorphous CaCO_3_ (precursor to calcite/aragonite shells) is utilised [275–277].

Crustacean species’ ability to deal with increasing OA also depends on life-history strategies and habitat [278]. Active species or those in highly fluctuating environments (*e.g.* intertidal or estuarine) tend to utilise the oxygen-transporting protein haemocyanin, which also confers additional buffering capacity against high H^+^ concentrations. Sedentary species or those in stable environments (*e.g.* deep-sea or polar) tend to have less haemocyanin and consequently less buffering capacity. The latter group relies more on ${\mathrm{HCO}}_{3}^{-}$ buffering and is probably more sensitive to OA.[278]

Recent studies on Alaskan King Crab (AKC, *Paralithodes camtschaticus*) and Tanner Crab (TC, *Chionoecetes bairdi*) in Alaskan waters highlight the vulnerability of the early life stages to OA [279, 280]. For AKC embryos and larvae, OA produces larger embryos (but not larger mass), smaller egg yolks, higher developmental rates, and higher calcium content [280]. In juveniles of both species, increased mortality occurs with elevated *P*_CO2_ (Table 1), with 100% mortality in their most extreme treatment (S2 Table) [279]. Differences between the two Alaskan crabs (decreased condition index in AKC but not TC and decreased calcium content in TC but not AKC) suggest that AKC puts more energy into osmoregulation and calcification than does TC [279]. Additionally, there is some preliminary evidence that adult AKC females fail to moult [280].

Initial studies are underway on the dominant local species of krill, *Euphausia pacifica*. A recent study in Puget Sound, WA (Fig. 1), found that elevated *P*_CO2_ slowed the development of hatched nauplii to the first feeding stage (Anna McLaskey, pers. comm., University of Alaska, Fairbanks AK). Also, under higher *P*_CO2_ the Antarctic krill species, *Euphausia superba*, experiences ingestion rates 3.5 times higher than those under present-day conditions, and consistently higher metabolic rates [281].

For the cold-water barnacle, *Semibalanus balanoides* (common in BC), experimental treatments at elevated CO_2_ (S2 Table) reduced adult survival and slowed embryonic development, which delayed the time of hatching by 19 days [282]. The cold-water shrimp, *Pandalus borealis* (common and commercially important in BC), also exhibited delayed juvenile development at reduced pH [283]. Other studies find no such delays [284–286], though significant effects have been observed when temperature and *P*_CO2_ interact [285]. The ability to tolerate OA also depends in part on prior exposure to habitats that experience highly fluctuating *P*_CO2_ [287].

**Indirect effects** Slow embryonic development [282] could potentially cause a timing mismatch between larval release and prey availability related to the spring phytoplankton bloom [288]. Potentially slower growth and lower fitness in juveniles and young adults may reduce egg production by females over their lifetime [279]. Despite the stability of adult exoskeletons, the post-moult calcification stage in crustaceans may be delayed significantly under elevated *P*_CO2_ [278], which may increase mortality due to predation on this defenseless life stage (*e.g.* [289]). Additionally, Kunkel *et al.* [290] hypothesise that OA may degrade the thin outer layer of calcite, which helps protect decapods from microbial attack. Finally, stock assessment models that incorporate reduced recruitment survival as a function of OA suggest that there can be a substantial socio-economic cost that is currently not recognised by decision makers [291].

**Crustacean synopsis** Generally, the crustaceans are expected to be sensitive to OA effects at early life cycle stages, while available studies suggest mixed results for adults. However, many local species, such as prawns, have not been studied (Fig. 4A). There is evidence that developmental anomalies in embryos and larvae occur at reduced pH, which may affect the fitness of juveniles and adults; however, the effects are species-specific and phenotypic adaptation is not known. Additionally, changes in growth rate and calcification may increase the susceptibility to predation, and delays in development may decouple life cycle timing between larval release and optimal foraging conditions.

### Fish

In BC coastal waters, there are over 300 species of marine fish [292, 293]. The taxonomic groups represented in BC include jawless fish (*e.g.* hagfish (270–1010 m)), cartilaginous fish (*e.g.* ratfish (50–380 m), dogfish (50–430 m), sharks (90–1020 m), skates (50–860 m)), and bony fish. The latter group includes important contributors to BC fisheries—Pacific Herring (*Clupea pallasi*, 5–170 m), salmon (five species of *Oncorhynchus*, mostly in the surface 50 m but some species deeper than 100 m), Pacific Hake (*Merluccius productus*, 80–700 m), Pacific Cod (*Gadus macrocephalus*, 50–300 m), Walleye Pollock (*Theragra chalcogramma*, 50–300 m), rockfish (at least 36 species of *Sebastes* (70–470 m) and two species of *Sebastolobus* (160–1010 m)), Sablefish (*Anoplopoma fimbria*, 70–970 m), Lingcod (*Ophiodon elongatus*, 50–310 m), Arrowtooth Flounder (*Atheresthes stomias*, 60–600 m), soles and flounders (∼18 species, 50–860 m), and Pacific Halibut (*Hippoglossus stenolepis*, 50–490 m). Depth distributions for valuable BC fisheries (Fig. 4) appear in Fig. 3. Marine fish species are economically important (GDP of capture fisheries, aquaculture, and sport fishing in BC was over $340 million in 2011 [29]) and ecologically valuable because of their roles providing food sources to higher trophic levels (*e.g.* birds and mammals) and cycling nutrients to other ecosystems (*e.g.* salmon providing nutrients to coastal terrestrial ecosystems [294]).

**Direct Effects** In general, we expect that adult fish will be tolerant of OA because they can control ion concentrations through evolved regulatory mechanisms [295, 296]. In particular, active fish exhibit transient elevated metabolic rates and highly variable extracellular CO_2_ and proton concentrations. Acid-base imbalances are regulated by specialised gill epithelia, which compensate for pH disturbances caused by exposure to increased environmental *P*_CO2_ [296]. Although some studies suggest that aerobic performance of tropical fishes may decline under elevated *P*_CO2_ [297] (Table 1), detrimental effects were not found in a temperate species, Atlantic Cod, under elevated *P*_CO2_ (*e.g.* [295]).

The effects of lake acidification on diadromous fish (those migrating between marine and fresh water) are well known, but using these observations to suggest OA effects is potentially misleading due to (i) large physiochemical differences between fresh and acidified marine waters and (ii) high physiological variability between diadromous and marine species [298, 299]. Also, fluctuations in in [H^+^] seen in lake acidification are orders of magnitude greater than those in the ocean [298].

As with the invertebrates, OA effects in fish are expected to occur during the vulnerable developmental stage, and these effects appear to be species specific. The acid-base regulatory mechanisms of the larval stage remain rudimentary until gills have formed and respiration switches from cutaneous to branchial [300]. Developmental responses are thought to be more the result of CO_2_ toxicity rather than through pH acting alone [301, 302].

There are limited OA studies on fish species that occur in our region. Hurst *et al.* [303] showed that the effects of OA on the growth of Walleye Pollock larvae were minor and varied greatly within treatments (S2 Table, Fig. 4). Slightly higher growth rates in elevated *P*_CO2_ conditions (Table 1) proved non-significant. Other studies on Atlantic temperate fish species (cod and herring), closely related to those in BC waters, found no significant effects on sperm motility, embryogenesis, egg survival, or the development of skeletal, heart, and lung tissue [300, 304, 305]. Despite these benign effects, researchers have found some developmental anomalies. Franke and Clemmesen [305] showed an inverse relationship for Atlantic Herring between *P*_CO2_ and the ratio RNA/DNA at hatching, potentially reducing protein biosynthesis and growth. Frommel *et al.* [300] found significant tissue damage in liver, pancreas, kidney, eye, and gut of Atlantic Cod larvae under elevated *P*_CO2_ Baumann *et al.* [306] demonstrated that increasing *P*_CO2_ caused a 74% reduction in survival and an 18% reduction in length of embryos of a ubiquitous estuarine fish called Inland Silverside (*Menidia beryllina*). Any significant developmental effect could alter the abundance and diversity of marine fish populations.

Otoliths (ear bones) are aragonite-based structures that fish use to sense acceleration and orientation. In some species, otoliths grow larger when larval fish are exposed to elevated *P*_CO2_ (*e.g.* White Sea Bass, a species found in BC waters [307]; Atlantic Cod [308] and tropical clownfish [309]). Under elevated *P*_CO2_ pH is regulated in the endolymph sac surrounding the otolith resulting in increased CaCO_3_ precipitation and enhanced otolith growth for those species [309]. An increase in otolith size may enhance hearing range [310], which might help or harm fish depending on sensitivity to important auditory cues or disruptive background noise [310].

Behavioural responses have recently been documented at elevated *P*_CO2_ for larvae of tropical reef fish. In particular, behaviour to olfactory, auditory, and visual cues changes when larvae are selecting habitats and responding to predators [311–315]. Additionally, elevated *P*_CO2_ reduces learning abilities related to predator avoidance [316] and changes the propensity of larval reef fish to turn left or right (lateralisation) [317]. These behavioural changes can expose larval fish to increased mortality risk, which has important fitness consequences [313, 318]. Given possible behavioural effects on predators as well as prey under elevated *P*_CO2_ community-level responses are difficult to predict [318, 319].

Relatively few studies have investigated behavioural changes to OA in temperate species (three exceptions being [320–322]), and none have examined commercially important species in BC waters. The larvae of Threespine Stickleback (*Gasterosteus aculeatus*), a species found in marine and fresh water on the BC coast, exhibit behavioural disturbances (*e.g.* reduction in boldness and curiosity), compromised learning abilities, and declines in lateralisation when reared in elevated *P*_CO2_ [320]. These responses are surprising given the physiological plasticity of this species, which is expected to confer enhanced acclimatisation abilities to environmental challenges. These results suggest that sensitivity to OA is not limited to species occupying narrow ecological niches, such as tropical reef fish [320].

Elevated *P*_CO2_ can disrupt the functioning of GABA_*A*_ (*γ*-Aminobutyric acid) receptors, the main inhibitory neurotransmitter receptors in the fish brain [323]. Normally, the opening of these receptors results in an inflow of Cl^−^ and ${\mathrm{HCO}}_{3}^{-}$ ions over the neuronal membrane, leading to inhibition of the neuron. When concentrations of intracellular Cl^−^ and ${\mathrm{HCO}}_{3}^{-}$are altered (e.g, when fish with strong acid-base regulatory systems are exposed to higher environmental *P*_CO2_, the flow of ions can be reversed, resulting in neuronal excitation instead of inhibition. Such changes have been associated with dramatic shifts in behaviour and sensory preferences in larval tropical reef fish [323], but the effects on temperate species are unknown. Although these receptors are shared by many, if not most fish, the resulting behavioural responses will likely vary due to species-specific differences in acid-base regulatory systems [323].

**Indirect effects** Fish will likely be affected indirectly by OA through food-web interactions. Off the southern WCVI, the pelagic system is dominated by Pacific Hake, Pacific Herring, Spiny Dogfish, and Chinook Salmon (*Oncorhynchus tshawytscha*), all largely dependent on krill production in the region [324]. This area has also been described as a “toxic hot spot” due to consistently high levels of *Pseudo-nitzschia* species and the presence of domoic acid [325]. These neurotoxins are transferred to higher trophic levels [59], and as *P*_CO2_ increases under OA the toxicity of these blooms may also increase [93].

Many fish species of the north Pacific Ocean prey on shelled pteropods (*e.g.* cod, pollock, mackerel) and a decline in pteropod abundances may lead to a shift in diet toward greater predation on juvenile fish such as salmon [326]. Pteropods (see Pteropods—Indirect effects) are also an important food source for Pink Salmon in the first year of marine life [166]. Because pteropods often exhibit swarming behaviour, foraging costs are relatively low for Pink Salmon feeding on patches [166, 180], possibly enhancing growth in early marine life and increasing adult biomass [327]. Reductions in pteropod densities may therefore have significant impacts on Pink Salmon biomass (Fig. 4A).

Trophodynamic modelling can suggest possible impacts of OA on fish populations. One study [328] explored various scenarios under OA, one of which assumes a significant mortality on benthic shelled invertebrates (*e.g.* bivalves, corals, sea urchins, sea stars) that leads to a biomass reduction for fish that feed on these species. While both English Sole (*Parophrys vetulus*) and small demersal sharks (*e.g.* Spiny Dogfish) rely on these invertebrates for only 10% of their diet in the model, English Sole experiences a much bigger decline due to a lack of alternative prey items. Another OA scenario in [328] adds an additional mortality on large zooplankton and small phytoplankton, which leads to a large increase in microzooplankton, detritus, and bacteria. In this scenario, the model predicts various higher-order interactions: a reduction of Lingcod due to a decline in macrozooplanktonic prey; an increase in Canary Rockfish (*Sebastes pinniger*) due to an increase in sea urchins and shrimps; and the increase of nearshore rockfish due to a decline in one of its predators, Lingcod. While there are many possible outcomes using such modelling tools, they do highlight how effects from OA on any single biological component can affect the entire trophic web.

**Fish synopsis** In general, we expect that adult fish will be tolerant of OA because of their ability to control internal ion concentrations. However, OA may affect fish during vulnerable developmental stages, though evidence for these effects is weak for species in BC. Perhaps more importantly, behavioural responses to OA have been widely documented in tropical reef fish, resulting in reduced survival. Similar effects may occur in temperate species, though studies in this area are limited. OA-induced reductions in availability of some prey species may reduce fish growth and survival, though these effects may be tempered by prey-switching. Possible increases in HABs would have a negative impact on farmed fish and shellfish; wild fish might increasingly suffer the effects of biotoxin accumulation.

### Marine mammals

British Columbia is host to a large and diverse group of marine mammals (∼30 species [329]), many of which have experienced dramatic population increases over the last century when hunting and culling practices were discontinued (*e.g.* on Grey Whales (*Eschrichtius robustus*) and Harbour Seals (*Phoca vitulina*), respectively) [330]. In addition to their role as top predator in the marine food web and their contribution to ecotourism, these mammals are iconic symbols of the region. Thus, they are valuable, but their value is difficult to assess (*e.g.* [331]).

In general, marine mammals cover an appreciable geographic range and many are able to dive to remarkable depths [332]. Their physiology is adapted to high pressures and they have an exceptional capacity for O_2_ [332]. Because they breathe at the surface, they are not susceptible to acidosis in the way that many other complex marine organisms will be as carbon levels increase (*e.g.* [302]). Therefore, direct impacts of OA on marine mammals are not expected, and have not been investigated (Fig. 4A). Indirect food web impacts are anticipated, *e.g.* for cetaceans that rely heavily on cephalopods or zooplankton such as pteropods [333]. In addition, underwater sound absorption at low frequencies (relevant for marine mammals) will decrease with OA [334]. However, this decrease is projected to be small (less than 0.2 dB) over the next few centuries and negligible in the context of the current noise associated with shipping [335].

**Marine mammal synopsis** Marine mammals will likely be affected by OA indirectly through food web changes, however direct impacts are not anticipated. While noise levels will increase with OA, this increase will not be large enough over the next few centuries to affect animals that rely on underwater sound.

## Discussion

We have described the marine ecosystem in the temperate coastal northeast Pacific region at present, and then its response to OA. However, the available information is limited. For some organisms, no OA studies exist (*e.g.* Geoduck Clam, rockfish, S1 Table). In general there are more studies, with respect to distributions and OA impacts, on species that are easier to observe, are of commercial value (*e.g.* oysters, S1 Table) or that threaten human health (*e.g.* harmful algae, S1 Table). The results of studies like these are often adopted when similar research on native organisms is not available (as we have done), limiting the ability to predict responses with confidence. Furthermore, OA is only one aspect of climate change and predicting shifts in marine ecosystems, and the degree to which they are caused by natural or anthropogenic forcing, is a highly complex problem. In the following, we discuss these and other issues that influence our evaluation.

**Caveats** The number of studies related to OA is growing rapidly (*e.g.* S1 Table). While experiments in these studies are highly valuable, translating their results into changes in the real world is challenging. For example, wild populations of marine organisms will adapt (both physiologically in a single lifespan and genetically over multiple generations) to their changing environment, which is difficult or impossible to capture *in vitro*. However, using temperature-dependent adaptation as a guide, Kelly and Hofmann [336] caution that the ability to adapt to changing pH may be limited.

In addition, food-web interactions and responses to OA are extremely difficult to predict, but will influence marine populations and could tip the balance from an overall negative impact to a positive one for a given species if a key predator is removed. Ecosystem effects resulting from OA have previously been identified as a key knowledge gap [337]. Furthermore, different life stages, particularly the juvenile stage (*e.g.* echinoderms), often display increased susceptibility to OA, but the impact of exposure of one life stage to low pH conditions on the subsequent life stages has only rarely been studied (but see [206, 212]). Similarly, even in organisms that have been comparatively well studied, not all life stages have been considered and certainly not within the context of the variability in natural conditions (Fig. 2).

Manipulated experiments generally consider present-day atmospheric conditions (∼360–400 *μ*atm) to be the control *P*_CO2_ level and all treatments above that to be ‘elevated’. Meanwhile *P*_CO2_ varies significantly with depth, and is naturally high in the north Pacific [28]. We quantify ‘elevated’ based on the local *P*_CO2_ levels at the depths of the organisms in question (Table 1). The combined effect of coastal upwelling, and local remineralisation of high production [27], results in exceptionally high (and variable) subsurface *P*_CO2_ on the outer BC shelf (Fig. 2). In local and connected inshore waters subsurface *P*_CO2_ is also high (unpublished data, DI; [6, 31]). Thus, many marine organisms in our region are currently experiencing conditions that are viewed as ‘elevated’ in the literature (Fig. 2; S2 Table). In addition, laboratory treatments often specify environmental conditions (*e.g.* temperature, *P*_CO2_) that do not occur in nature and are unlikely to occur, at least locally (*e.g.* [31]). Exposure time may also limit the interpretation of results, as there are distinct differences between treatments that are ‘shocked’ and those that are allowed to acclimate (*e.g.* [87, 248]).

Finally, defining the carbon state in seawater is not trivial [10] and requires that at least two of the four carbon parameters (DIC, TA, *P*_CO2_, pH) be measured. The quality of the measurements and manipulation in the laboratory work cited here is variable. While the high degree of accuracy and precision required by chemical oceanographers [10] is in general not necessary to obtain insight from biological manipulation experiments, the equations that define the carbon system lead to compounding errors when calculating one of the unknowns. Thus, a moderate uncertainty in *P*_CO2_ may translate to an estimated pH that has little, or no meaning. We urge the reader to consult S2 Table where all available detail for each experiment cited has been summarised.

**Climate change—the whole picture** The ocean has absorbed a significant portion of the anthropogenically produced carbon [2] and that has caused on average a 30% change in surface ocean acidity [5]. However the annual variability in surface *P*_CO2_ and pH in dynamic regions like the BC [24] and WA [31] coasts is generally more than two orders of magnitude greater than the annual atmospheric increase in CO_2_. In other words, we expect the OA trend to be present, but overlaid is a signal with large amplitude.

Climate change may alter this dynamic natural cycle so that negative impacts associated with high acidity are experienced earlier in the coastal northeast Pacific than elsewhere, regardless of OA. There are critical times during the year when carbon conditions (particularly in the upper mixed layer; 20–30 m on the outer coast; ∼10 m or less in protected waterways) change dramatically. For example, the spring bloom in the Strait of Georgia causes a large and rapid increase in surface pH (Ben Moore Maley pers. comm., University of British Columbia, Vancouver BC) and the timing of this event varies significantly from year to year [338]. On the outer shelf, the onset of summer upwelling brings lower pH water over the continental shelf and decreases pH (on average) throughout the entire water column. Climate change may alter the strength, timing [339–341], or even the variability in the timing, of such events. Thus, the influence of climate change on weather may play a critical role, that will only be exacerbated as OA progresses.

In addition to changing weather, sea surface temperatures are expected to increase and subsurface O_2_ is expected to decrease (leading to increased occurrence of hypoxia) with climate change, concurrent with OA. Temperature has a large effect on marine organisms because metabolism increases as the ocean warms, consequently increasing energetic costs. As a result, changes in present-day distributions of marine organisms have already been linked to changes in temperature [342]. Thus, a ‘multi-stressor’ approach is required to understand the net effect of climate change on marine organisms. The net effect of all three stressors (warming, hypoxia and OA) may be synergistic and has been generally described as a narrowing of the thermal ranges in which organisms can perform well, and a decrease in maximal performance [343]. Lastly, changes in human behaviour (*e.g.* fishing) as climate change and OA progress may also play an important, and possibly additive, role in shaping future marine ecosystems (*e.g.* [344]).

## Conclusions

There remain significant knowledge gaps with respect to the biological impacts of OA on marine ecosystems globally, and locally. The most critical impacts will likely be indirect as a result of food web changes, and so are highly complex and difficult to predict even with extensive study. Furthermore, OA related changes will occur in concert with other climate change impacts that may be even more severe (see above). In particular, increasing temperature and decreasing dissolved oxygen are likely to produce synergistic effects.

The northeast Pacific region naturally has waters low in pH (undersaturated with respect to aragonite) near the surface. Thus, it is potentially more vulnerable to OA than other regions. We summarise the most relevant risks and identify key knowledge gaps, given present-day knowledge, to Pacific Canadian fisheries and marine ecosystems in the order of immediacy and certainty.

- Shellfish aquaculture is highly susceptible to OA due to the direct impact of OA on shell formation and the dependence of the industry on hatchery production. These impacts are already experienced in BC (and WA). Wild shellfish experience similar difficulties but have the opportunity to adapt (*e.g.* [197]) and so will likely not be affected as rapidly and severely.
- There are no studies on Geoduck Clams, which are responsible for a lucrative wild fishery and a growing aquaculture industry in BC (although the latter is still in its infancy).
- The commercial BC fishery is dominated monetarily by salmon aquaculture. While uncertainty remains low, it is anticipated that the fish-killing alga *Heterosigma akashiwo* will gain a competitive advantage under OA, making blooms more frequent. Such blooms are already a significant issue for this industry in BC.
- Neurotoxins produced by other harmful algae are expected to become more potent under OA. Such blooms already cause shellfish closures in BC. If this increase in toxicity occurs, the shellfish industry will be affected. In addition, these toxins may cause decreased reproductive success, and even mass mortality, at higher trophic levels including fish, seabirds and marine mammals.
- Food web changes due to OA (*e.g.* in BC changes in the species composition of phytoplankton and decline of pteropods) are anticipated but remain unknown, as are the impacts of these lower level changes on higher trophic levels.
- Finfish are likely to experience OA impacts through foodweb changes. In BC examples include: the decline of pteropods, that are directly preyed upon by some fish (particularly Pink Salmon), and the anticipated decline of some echinoderms, that are eaten by various species of rockfish and flatfish.
- Habitat changes may also have a critical negative impact, in particular for juvenile fish. While these impacts remain highly uncertain, there may be a shift from upright macroalgae to algal turf. Also, local coral species (in BC primarily octocorals) that provide vertical structure may decline. Direct impacts of OA on finfish may also occur, but only at relatively high levels of CO_2_.
- There are few direct OA studies on local finfish species and none on Pacific Halibut and salmon, which drive the sport fishing industry. Similarly there are no studies on the adaptation of these local species to OA and multiple stressors, like temperature and O_2_, that will be changing at the same time. Because sport fishing dominates fishery related income in BC, this knowledge gap is significant.
- Behavioural changes at various trophic levels have been observed (*e.g.* increased downward swimming in phytoflagellates, decreased detection and avoidance of predators in larval fish) and postulated (*e.g.* increased movement to OA refugia such as eelgrass meadows). Such behavioural changes might alter the structure of marine communities in BC, and present another knowledge gap.
- Crabs may experience negative impacts under OA while other crustaceans significant to the harvest fishery in BC, like prawns, have not been well studied but appear to be more strongly sensitive to temperature than OA. In general, the juvenile stages of crustaceans are most vulnerable to OA, growing more slowly because they need to expend more energy under OA.

## Supporting Information

S1 Table Number of articles by Group of Organisms. Number of hits by Web of Science for OA-related studies on different groups of animals in March 2013 and March 2014.
(PDF)

S2 Table Experimental details for manipulation experiments. The experimental details for all manipulation experiments referred to in this document.
(PDF)

S3 Table Environmental details for *in situ* studies. Details (e.g. species, location, and carbon state) for OA-related *in situ* studies of marine organisms.
(PDF)

S1 Text Pacific Region Animal Use Protocols. Marine animal use protocols in British Columbia, Canada.
(PDF)

S1 Data Species depth distribution data. The data required to generate Fig. 3.
(XLSX)

S1 Code R-code to create Fig. 1. The header within the code contains necessary instructions.
(R) Click here for additional data file.

S2 Code R-code to create Fig. 2. The code includes the data required to generate the figure.
(R) Click here for additional data file.

S3 Code R-code to create Fig. 3. The S1 data are required to generate this figure.
(R) Click here for additional data file.

S4 Code R-code to create Fig. 4. The code includes the data required to generate the figure.
(R) Click here for additional data file.
