# Supplementary material for: Effects of Ocean Acidification on Temperate Coastal Marine Ecosystems and Fisheries in the Northeast Pacific
Source: PLoS One. 2015 Feb 11;10(2):e0117533. doi: 10.1371/journal.pone.0117533 (PMC4324998; doi:10.1371/journal.pone.0117533)
Supplement: S1 Table — Number of hits by Web of Science for OA-related studies on different groups of animals in March 2013 and March 2014. (PDF) [file pone.0117533.s001.pdf]

Supplemental Tables

Table S1. The number of articles (peer-reviewed papers, reviews and book chapters) for each search string as given by Web of Science on 28th March 2013 and 28th March 2014. Search strings are (Primary keyword) AND (Secondary keyword). The secondary keywords are listed in the first column. The primary keyword is (“Ocean Acidification” OR pH OR PCO2 OR CO2 OR “Carbon Dioxide”) for the first two columns of results (to include papers that may have not explicitly mentioned the term “Ocean Acidification”), and just “Ocean Acidification” for the final two columns. Keywords are located in the following Web of Science fields: Title, Abstract, Keywords and Keywords Plus® (relevant keywords within reference list of selected paper). A * indicates any character(s), with examples given in (·).

	“Ocean Acidification” OR ...		“Ocean Acidification”	
Secondary keyword	March 2013	March 2014	March 2013	March 2014
(no secondary keyword)	682,741	740,027	1,133	1,709
“North Pacific”	638	708	30	42
Phytoplankton	3,304	3,673	159	235
Alga* (-e, -l)	6,879	7,682	152	244
“Harmful alga* bloom*” (-e, -l, -s)	80	101	17	24
Diatom* (-s)	2,078	2,830	37	53
Coccolith* (-ophore, -ophorid)	298	371	82	105
Macroalga* (-e, -l)	548	635	49	84
Seagrass* (-es)	221	246	28	39
Zooplankton	847	924	29	45
Copepod* (-s)	336	372	16	28
Krill	83	89	3	6
Euphausi* (-a, -id)	65	69	3	6
Pteropod* (-s)	54	62	33	41
Shellfish* (-es)	260	292	17	24
Mollus* (-c, -ca, -k)	826	902	67	97
Geoduck* (-s)	2	2	0	0
Clam* (-s)	3,082	3,202	16	27

Continued on next page

Table S1 – continued from previous page

	"Ocean Acidification" OR ...		"Ocean Acidification"	
Secondary keyword	March 2013	March 2014	March 2013	March 2014
Oyster* (-s)	453	554	50	83
Scallop* (-s)	173	187	7	14
Mussel* (-s)	712	814	45	68
"Cold-water coral*" (-s)	24	40	22	35
"Glass sponge*" (-s)	1	1	0	0
Benth* (-ic, -os)	1,984	2,233	151	221
Invertebrate* (-s)	1,620	1,780	143	195
Echinoderm* (-ata, -s)	133	152	53	66
"Sea cucumber*" (-s)	58	65	3	3
"Sea urchin*" (-s)	632	680	95	129
Crab* (-s)	915	961	38	50
Shrimp* (-s)	1,189	1,286	5	8
Prawn* (-s)	290	295	3	4
Fish* (-es, -ies, -y)	9,441	10,207	159	238
Herring	226	240	2	2
Sardine* (-s)	240	251	0	1
Groundfish* (-es)	2	2	1	1
Flatfish* (-es)	32	33	0	0
Rockfish* (-es)	16	16	1	1
Squid* (-s)	636	664	7	11
Octopus	92	96	3	4
Salmon	1,456	1,559	7	12
(Sea OR marine) AND bird* (-s)	79	85	4	6
Mammal* (-s)	7,177	7,550	10	13
Whale* (-s)	376	389	4	5
Elasmobranch* (-s)	105	107	2	2
Seal* (-s)	2,535	2,691	2	6
Shark* (-s)	201	211	4	4
