# Supplementary material for: Effects of Ocean Acidification on Temperate Coastal Marine Ecosystems and Fisheries in the Northeast Pacific
Source: PLoS One. 2015 Feb 11;10(2):e0117533. doi: 10.1371/journal.pone.0117533 (PMC4324998; doi:10.1371/journal.pone.0117533)
Supplement: S2 Table — The experimental details for all manipulation experiments referred to in this document. (PDF) [file pone.0117533.s002.pdf]

Table S2. Experimental details for references in the paper. P_{CO2} is presented in units of μatm. The control variable (usually P_{CO2}) in each experiment is reported based on the manipulation (*e.g.* the P_{CO2} of air in each treatment using the bubbling technique). The carbonate parameters measured within the seawater treatments (regardless of method of manipulation and control variable) are reported. Exposure time: s = seconds, min = minutes, h = hours, d = days, hpf = hours post fertilisation, dpf = days post fertilisation, dph = days post hatching, w = weeks, mo = months, y = years, g = generations. Other detail: S = salinity, I = irradiance, γ = photons, L:D = light:dark regime, T = temperature. n.r. = not reported or not measured.

Reference	Species	Control variable	Expt.s	Measured carbon	Exposure time	Other detail
**Phytoplankton**						
Bach *et al.* 2011 [20]	*Emiliania huxleyi* B92/11	P_{CO2}	20-6000; 40-3700	TA, pH, DIC	> 1 d	15°C
Bellerby *et al.* 2008 [77]	natural assemblage (mesososm, Bergen)	P_{CO2}	350, 700, 1050	P_{CO2}, TA, DIC	25 d	
Burkhardt *et al.* 1999 [83]	*Asterionella glacialis*, *Thalassiosira punctigera*, *Coscinodiscus wailesii*, *Phaeodactylum tricornutum*, *Scrippsiella trochoidea*	$[CO_2]$ (μmol/kg)	1-38	DIC, TA	n.r.	15°C, L:D=16:8/24:0, I=150 μmol γ m^{-2}s^{-1}
Clark & Flynn 2000 [81]	*Phaeodactylum tricornutum*, *Thalassiosira pseudonana*, *Thalassiosira weissflogii*, *Stichococcocus bacillaris*, *Alexandrium fundyense*, *Scrippsiella trochoidea*, *Emiliania huxleyi*, *Isochrysis galbana*, *Heterosigma carterae*	DIC (mM)	0.25, 0.5, 1, 2	DIC, pH	2 growth cycles	16°C, L:D=12:12, I=200 μmol γ m^{-2}s^{-1}
Delille *et al.* 2005 [100]	natural assemblage (mesocosm, Bergen)	P_{CO2}	180, 370, 700	TA, P_{CO2}	25 d	
Egge *et al.* 2009 [78]	natural assemblage (mesocosm, Bergen)	P_{CO2}	180, 370, 700<br>190, 370, 700<br>375, 750, 1150	n.r.	25 d<br>25 d<br>25 d	10-13°C<br>8-10°C<br>9-11.5°C
Engel *et al.* 2005 [67]	*Emiliania huxleyi* (mesocosm, Bergen)	P_{CO2}	190, 410, 710	P_{CO2}, TA, pH	19 d	10-13°C, $\bar{I}$=598 μmol γ m^{-2}s^{-1}
Fu *et al.* 2008 [82]	*Heterosigma akashiwo* CCMP2393, *Prorocentrum minimum* CCMP2233	P_{CO2}	375, 750	pH, DIC	n.r.	20°C, 24°C
Hare *et al.* 2007 [85]	natural assemblage (Bering Sea)	P_{CO2}	370, 750	n.r.	9-10 d	10.4°C, 15°C
Hein & Sand-Jensen 1997 [79]	natural assemblage (Atlantic transect)	$[CO_2]$ (μM)	3, 10, 36, 91	n.r.	2 h	ambient T (n.r.)
Hwang & Lu 2000 [89]	*Alexandrium minutum*	pH	5.5, 7.5, 8.5	pH	36 d	
Kim *et al.* 2013 [87]	*Heterosigma akashiwo*	P_{CO2}	280, 380, 750	pH, DIC, TA	7 d	15°C, L:D=12:12, I=70-80 μmol γ m^{-2}s^{-1}
Nielsen *et al.* 2010 [68]	natural assemblage (microcosm, Denmark)	pH	6, 7.6, 7.8, 8	pH, DIC	14 d	May (10.7°C, S=18.7), Aug (17.9°C, S=10.0)

Continued on next page

Table S2 – continued from previous page

Reference	Species	Control variable	Expt.s	Measured carbon	Exposure time	Other detail
Nimer *et al.* 1977 [76]	*Skeletonema costatum*, *Chaetoceros compressus*, *Melosira* sp., *Thalassiosira rotula*, *T. weisfloggi*, *T. pseudonana*, *Phaeodactylum tricornutum*, *Scrippsiella trochoidea*, *Amphidinium carterae*, *Prorocentrum micans*, *P. minimum*, *Glenodinium foliaceum*, *Chrysochromulina kappa*, *Gephyrocapsa oceanica*, *Coccolithus pelagicus*, *Pleurochrysis carterae*, *Emiliania huxleyi*, *Heterosigma akashiwo*	DIC (mM)	1, 2	TA, pH	10 d	15°C, I=200 μmol γ m^{-2}s^{-1}
Riebesell *et al.* 2000 [70]	*Emiliania huxleyi* (PML B92/11A), *Gephyrocapsa oceanica* (PC7/1)	P_{CO2}	280-750	DIC, TA	7-9 g	15°C, S=31, L:D=(16:8, 24:0), I=30-150 μmol γ m^{-2}s^{-1}
Riebesell *et al.* 2007 [80]	natural assemblage (mesocosm, Raunefjord, Norway)	P_{CO2}	350, 700 1050	P_{CO2}, TA, DIC	24 d	95% light transmission
Shi *et al.* 2009 [97]	*Thalassiosira weissflogii* (CCMP 1336), *Emiliania huxleyi* (CCMP 374, PLY M219)	P_{CO2}<br>pH	380, 750<br>7.8, 8.1	DIC, TA, pH	4 d	20°C, L:D=24:0, I=150 μmol γ m^{-2}s^{-1}
Sun *et al.* 2011 [92]	*Pseudo-nitzschia multiseries*	P_{CO2}	220, 400, 730	pH, DIC	4-6 w	17°C
Tatters *et al.* 2012 [93]	*Pseudo-nitzschia fraudulenta* USCWWA7	P_{CO2}	200, 360, 765	DIC, pH	4 min	16°C
Tatters *et al.* 2013 [91]	*Alexandrium catenella*	P_{CO2}	380, 750	DIC, pH	8 mo	T=14,19°C, 0.4,2 μm PO_4^{3-}
Tortell *et al.* 2002 [86]	natural assemblage equatorial Pacific (Peru)	P_{CO2}	150, 750	pH	3-8 d	on deck expt.
Tortell *et al.* 2008 [84]	natural assemblage	P_{CO2}	100, 380, 800	pH	10-18 d	ambient T ($\sim$ 1°C)
Zondervan *et al.* 2001 [98]	*Emiliania huxleyi* (strain PML B92/11) *Gephyrocapsa oceanica* (strain PC 7/1)	CO_2 (μmol L^{-1})	5.61, 12.36, 18.45, 21.32, 27.25	DIC, TA	12 g	15°C, L:D=16:8, 24:0, I=150 μmol γ m^{-2}s^{-1}
**Macroalgae**						
Asnaghi *et al.* 2013 [125]	*Corallina elongata*, *Cystoseira amentacea*, *Dictyota dichotoma*	P_{CO2}	390, 550, 750 1000	TA, pH	1 mo	24°C; expt incl. grazing by sea urchins, *Paracentrotus lividus*
Connell & Russell 2010 [131]	*Ecklonia radiata*, turf-forming algal species not specified	P_{CO2}	380, 550	TA, pH	14 w	T=17-20°C
Cornwall *et al.* 2012 [107]	*Corallina officinalis* L., *Rhodophyllis gunnii* Harv, *Schizoseris* sp., *Ulva* sp., *Undaria pinnatifida* Harv.	pH	7.5, 7.9	TA, pH	5 d	contrasted acid addition w/ bubbling, 12°C
Gao *et al.* 1991	*Porphyra yezoensis* Ueda	P_{CO2}	350, 1000, 1600	–	25 d	15°C
Gao & Zheng 2010 [119]	*Corallina sessilis*	P_{CO2}	380, 1000	pH,–	31 d	UV treatments
Hofmann *et al.* 2012 [113]	*Corallina officinalis*, *Chondrus crispus*	P_{CO2}	385, 665, 1486	pH, TA	86 d	

Continued on next page

Table S2 – continued from previous page

Reference	Species	Control variable	Expt.s	Measured carbon	Exposure time	Other detail
Hepburn *et al.* 2011	*Marginariella boryana*, *Carpophyllum flexuosum*, *Cystophora retroflexa*, *Macrocystis pyrifera*, *Ecklonia radiata*	none	variable	pH	24 h	pH drift experiments
Hurd *et al.* 2011 [116]	*Sporolithon durum*	pH	8.11, 7.50	pH	7 h	12°C
Israel & Hophy 2002	*Chlorophyta* sp., *Rhodophyta* sp., *Phaeophyta* sp.	P_{CO2}	ambient, 750	pH, –	5, 28-105 d	
Kubler *et al.* 1999 [109]	*Lomentaria articulata*	P_{CO2}	235, 350, 700, 1750	DIC, pH	21 d	10°C
Kuffner *et al.* 2008 [122]	Crustose coralline algae, various spp.	pH	7.91-8.17	TA, pH	51 d	DIC not stored properly
Ries *et al.* 2009 [275]	*Halimeda incrassata*, *Neogoniolithon*	P_{CO2}	409, 606, 903, 2856	–	60 d	25°C, L:D=10:14, I=213-426 W/m^2
Roleda *et al.* 2012b [112]	*Macrocystis pyrifera*	pH	7.59-8.50	TA, pH	5, 43 d	UV treatments
Swanson & Fox 2007 [111]	*Saccharina latissima*, *Nereocystis luetkeana*	P_{CO2}	360, 3000	TA, pH	55 d	UV treatments
**Seagrass**						
Arnold *et al.* 2012 [140]	*Ruppia maritima*, *Potamogeton perfoliatus*	P_{CO2}	158-6792, 244-3465	P_{CO2}, pH	Maryland	two rivers of Chesapeake Bay
**Microzooplankton**						
Aberle *et al.* 2013 [148]	natural assemblage (mesocosm, Kongsfjorden, Svalbard)	P_{CO2}	175-250, 340-600, 675-1085	TA, pH	29 d	S=33.9
Kim *et al.* 2010 [151]	natural assemblage (mesocosm, coastal Korea)	P_{CO2}	400, 900	pH	20 d	ambient T(∼11-14°C), ambient + 3°C
Rose *et al.* 2009 [150]	natural assemblage (onboard, N. Atlantic)	P_{CO2}	390, 690	DIC, pH	14 d	T=12, 16°C
Rossol *et al.* 2013 [149]	natural assemblage (mesocosm, Kiel Fjord, Germany)	P_{CO2}	380, 840, 1120, 1400, 4000	pH, TA	28 d	18°C, S=18.1
Suffrian *et al.* 2008 [147]	natural assemblage (mesocosm, Raunefjord, Norway)	P_{CO2}	350, 700, 1050	n.r.	25 d	9-11.5°C
**Mesozooplankton**						
Kurihara *et al.* 2004 [157]	*Acartia steueri*, *A. erythraea*	P_{CO2}	365, 2365, 10365	P_{CO2}, pH	8 d	
Kurihara & Ishimatsu 2008 [159]	*Acartia tsuensis*	P_{CO2}	380, 2380	P_{CO2}, pH	9 d, 2 g ≃26 d	
Mayor *et al.* 2007 [158]	*Calanus finmarchicus*, *C. glacialis*	P_{CO2}	380, 8000	P_{CO2}, pH	5 d	
Mayor *et al.* 2012 [162]	*Calanus helgolandicus*	P_{CO2}	380, 1000	P_{CO2}, pH	3 d	8, 10, 12°C
Pedersen *et al.* 2013 [163]	*Calanus finmarchicus*	P_{CO2}	365, 3332, 7281, 9651	pH, TA	28 d	
Weydmann *et al.* 2012 [160]	*Calanus glacialis*	pH	6.9, 7.6, 8.2	pH	7 d	
Vehmaa *et al.* 2012 [161]	*Acartia bifilosa*	pH	7.39-8.13	pH	5 d + 3 d	17, 20°C
Zhang *et al.* 2011 [156]	*Acartia pacifica*, *A. spinicauda*, *Calanus sinicus*	P_{CO2}	380, 800, 2000, 5000, 10000	pH, TA	8 d	

Continued on next page

Reference	Species	Control variable	Expt.s	Measured carbon	Exposure time	Other detail
**Macrozooplankton – pteropods**						
Bednarsek *et al.* 2014	*Limacina helicina*	Ω_{arag}	% of water column saturated	DIC, TA	USA Pacific coast	0 – 200 m depth
Comeau *et al.* 2009 [175]	*Limacina helicina*	P_{CO2}	350, 760	TA, pH	2, 4, 6 h	5°C
Comeau *et al.* 2010 [176]	*Cavolinia inflexa*	pH	8.1, 7.8, 7.5	TA, pH	5, 13 d	13°C
Comeau *et al.* 2012 [177]	*Limacina helicina*	pH	8.05, 7.90, 7.75	pH, TA	8 d	-1.6°C
Lischka & Riebesell 2012 [170]	*Limacina helicina*, *Limacina retroversa*	P_{CO2}	350, 650 880	TA, pH	7 d	2, 7°C
Maas *et al.* 2012 [179]	*Hyalocyclis striata*, *Clio pyramidata*, *Cavolinia longirostris*, *Cresis virgula*, *Diacria quadridentata*	P_{CO2}	380, 1000	pH, –	6-18 h	20°C
Seibel *et al.* 2012 [178]	*Limacina helicina antarctica*	P_{CO2}	380, 790, 1000	TA, pH	4-13 d	-1.8°C
**Molluscs**						
Barros *et al.* 2013 [200]	*Crassostrea gigas*	pH	7.37, 7.76, 8.09	pH	2, 3, 4 hpf, 2, 3, 6 dpf	
Barton *et al.* 2012 [195]	*Crassostrea gigas*	–	pH 7.6-8.2	P_{CO2}	24 h, 8 d	natural hatchery conditions over 64 d
Bechmann *et al.* 2011 [283]	*Mytilus edulis*	pH	7.61, 8.09	pH, –	64 dpf	10°C
Clark *et al.* 2013	*Crassostrea gigas*	pH	7.5, 7.9	pH	3 mo	T=19, 24°C, molecular analyses
Crim *et al.* 2011 [204]	*Haliotis kamtschatkana*	P_{CO2}	400, 800, 1800	P_{CO2}, pH, TA	1-8 dpf	
Dineshram *et al.* 2012 [211]	*Crassostrea gigas*	pH	7.5, 8.0	pH, TA	4, 6 dpf	proteome analysis
Duckworth & Peterson 2013 [220]	*Argopecten irradians*, *Cliona celata*	pH	7.8, 8.1	pH	133 d	T=26, 31°C, sponges boring into dead scallop shells
Gazeau *et al.* 2007 [213]	*Crassostrea gigas*	P_{CO2}	698-2774	pH, TA	2 h	
Ginger *et al.* 2013 [203]	*Crassostrea gigas*	pH	7.43, 7.66, 8.03	pH, TA	40 d	
Gobler *et al.* 2014 [205]	*Argopecten irradians*	pH	Expt. 1: 7.53, 8.00 Expt. 2: 7.37 (ambient), 7.92	pH, DIC	30-40 d	Expt. 1: O_2 =37, 260μatm; Expt. 2: O_2 =140 (ambient), 260μatm
Havenhand & Schlegel 2009 [198]	*Crassostrea gigas*	pH	7.82, 8.15	pH, TA	2 min, 1 h	
Hettinger *et al.* 2012 [212]	*Ostrea lurida*	P_{CO2}	714-1355	pH, TA	varied	Expt. 1: 11-13 d (larvae) + 7 d (settled), 54 d (juveniles, control P_{CO2}); Expt. 2: 13 d (larvae), 13 d (juveniles, same/alternate P_{CO2})
Hettinger *et al.* 2013 [206]	*Ostrea lurida*	P_{CO2}	485, 1060	DIC, pH, TA	11 d	juveniles returned to wild, monitored 4 mo

Continued on next page

Table S2 – continued from previous page

Reference	Species	Control variable	Expt.s	Measured carbon	Exposure time	Other detail
Hettinger *et al.* 2013 [207]	*Ostrea lurida*	P_{CO2}	520, 1075	DIC, pH, TA	11 d	low/high food supply
Kaplan *et al.* 2013 [225]	*Doryteuthis pealeii*	P_{CO2}	390, 2200	pH, TA	20 d	20°C, S=30.8, L:D=12:12
Kurihara *et al.* 2007 [201]	*Crassostrea gigas*	P_{CO2}	348, 2268	pH, TA	2, 3, 8, 24, 48 h	
Lacoue-Labarthe *et al.* 2011 [224]	*Loligo vulgaris*	P_{CO2}	380, 850, 1500	pH, TA	eggshells 26 d paralarvae 52 h	16°C, L:D=12:12
Lannig *et al.* 2010 [216]	*Crassostrea gigas*	P_{CO2}	582, 1480	pH, TA	26-55 d	T=15, 20, 25°C
Mingliang *et al.* 2011 [214]	*Chlamys farreri*	pH	6.96, 7.28, 7.58, 7.87, 8.08	pH, TA, DIC	2 h	
O'Donnell *et al.* 2013 [215]	*Mytilus trossulus*	P_{CO2}	300, 500, 600, 800, 1000, 1100, 1200, 1300, 1500	pH, TA	20 d	10°C, Shellfish Diet 1800
Parker *et al.* 2010 [199]	*Crassostrea gigas*	P_{CO2}	375, 600, 750, 1000	pH, TA	2 h, 2 dpf, 4 d	T=10, 22, 26, 30°C
Ries *et al.* 2009 [275]	*Mercenaria mercenaria*, *Mytilus edulis*, *Mya arenaria*, *Argopecten irradians*, *Crassostrea virginica*	P_{CO2}	409, 606, 903, 2856	–	60 d	25°C, L:D=10:14, I=213-426 W/m^2
Sanders *et al.* 2013 [217]	*Pecten maximus*	P_{CO2}	414, 528, 866, 1256	pH, TA, DIC	82-84 d	RNA:DNA ratios
Schalkhausser *et al.* 2013 [218]	*Pecten maximus*	P_{CO2}	390, 1120	pH, DIC	33-60 d	T=4, 10°C
Talmage & Gobler 2009 [208]	*Argopecten irradians*	CO_2 (Pa)	39, 66, 152	pH, DIC	19 d	
Talmage & Gobler 2011 [209]	*Argopecten irradians*	P_{CO2}	larvae: 240, 387, 773 juveniles: 400, 1700	pH, DIC	larvae: 20 d juveniles: 45 d	T=24, 28°C
Talmage & Gobler 2012 [210]	*Argopecten irradians*	P_{CO2}	232, 373, 864	pH, DIC	20 d	low- & high-bloom brown tide alga, plus control food source
Timmins-Schiffman *et al.* 2013 [202]	*Crassostrea gigas*	P_{CO2}	468, 847, 1065	pH, TA	1, 3 dpf	
Waldbusser *et al.* 2013 [17]	*Crassostrea gigas*	P_{CO2}	364	pH, P_{CO2}	2-19 dpf	stable carbon isotope analysis
**Sponges & Cold-water Corals**						
Form & Riebesell 2012 [248]	*Lophelia pertusa*	P_{CO2}	509, 605, 856, 981	DIC, TA	8 d	3 mo acclimation
		P_{CO2}	604, 778, 982	DIC, TA	178 d	
Maier *et al.* 2009 [247]	*Lophelia pertusa*	pH	ambient (8.1) a-0.15, a-0.3	pH, –	1 d	acid addition
Ries *et al.* 2009 [275]	*Oculina arbuscula*	P_{CO2}	409-2856	–	60 d	25°C, L:D=10:14, I=213-426 W/m^2
**Echinoderms**						
Asnaghi *et al.* 2013 [125]	*Paracentrotus lividus*	P_{CO2}	390, 550, 750 1000	pH. TA	1 mo	24°C, fed calcifying & non-calcifying macroalgae

Continued on next page

Table S2 – continued from previous page

Reference	Species	Control variable	Expt.s	Measured carbon	Exposure time	Other detail
Asnaghi *et al.* 2014 [258]	*Paracentrotus lividus*	P_{CO2}	390, 550, 750 1000	pH, TA	1 mo	22°C, fed calcifying & non-calcifying macroalgae
Clark *et al.* 2009 [257]	*Tripneustes gratilla*, *Pseudechinus huttoni*, *Evechinus chloroticus*, *Sterechinus neumayeri*	pH	6.0, 6.5, 7.0, 7.5, 7.7, 7.8, 8.2	pH, –	4 d	T=-1, 10-15, 26°C
Dupont *et al.* 2008 [266]	*Ophiothrix fragilis*	pH	7.7, 7.9, 8.1	pH, TA	8 d	14°C, S=32
Dupont *et al.* 2013 [259]	*Strongylocentrotus droebachiensis*	P_{CO2}	400, 1200	pH, TA	4 or 16 mo	12°C, fed *Ulva*
Morita *et al.* 2010 [264]	*Holothuria* sp.	pH	6.6, 7.3, 7.6, 7.7, 7.8, 8.0	pH	2 s video footage of motility	27°C, animals held in pH treatment before sperm extraction
O'Donnell *et al.* 2009 [261]	*Strongylocentrotus franciscanus*	CO_2	380, 540, 970	pH, –	96 h	15-31°C
Pespeni *et al.* 2013 [263]	*Strongylocentrotus purpuratus*	P_{CO2}	400, 900	pH, TA	7 dpf	14°C
Reuter *et al.* 2011 [260]	*Strongylocentrotus franciscanus*	CO_2	400, 800, 1800	pH, TA	3 h	7-11°C
Todgham & Hofmann 2009 [262]	*Strongylocentrotus purpuratus*	P_{CO2}	380, 540, 1020	pH, –	28, 40, 79 dpf	15°C, O_2 ≥7.9mg/L
**Crustaceans**						
Bechmann *et al.* 2011 [283]	*Pandalus borealis*	pH	7.63, 8.13	pH, –	5 w	5°C
Findlay *et al.* 2009 [282]	*Semibalanus balanoides*	CO_2	344, 922	pH, DIC	104 d	10-13°C
Long *et al.* 2013a [279]	*Paralithodes camtschaticus*, *Chionoecetes bairdi*	pH	7.5, 7.8, 8.0	pH, DIC, TA	192, 199d,	local ambient T and S
Long *et al.* 2013b [280]	*Paralithodes camtschaticus*	pH	7.7, 8.0	pH, DIC	∼ 2 mo	4.1°C
Pansch *et al.* 2012 [285]	*Amphibalanus improvisus*	P_{CO2}	400, 1250, 3250	pH, DIC, TA	2 mo	T=12, 20, 27°C
Pansch *et al.* 2013 [286]	*Amphibalanus improvisus*	pH	7.6, 7.8, 8.0, 8.1	pH, TA	10 d	20°C
Pansch *et al.* 2014 [287]	*Amphibalanus improvisus*	P_{CO2}	400, 1000, 3000	pH, TA	20 w	10-20°C, altered food availability
Ries *et al.* 2009 [275]	*Callinectes sapidus*, *Homarus americanus*, *Penaeus plebejus*, *Littorina littorea*, *Urosalpinx cinerea*, *Crepidula fornicata*, *Strombus alatus*, *Eucidaris tribuloides*, *Arbacia punctulata*, *Hydroides crucigera*	P_{CO2}	409, 606, 903, 2856	–	60 d	25°C, L:D=10:14, I=213-426 W/m^2
Saba *et al.* 2012 [281]	*Euphausia superba*	P_{CO2}	452, 672	DIC, TA	n.r.	0.65°C, onboard incubations
**Fish – Behaviour**						
Bignami *et al.* 2013 [310]	*Rachycentron canadum*	pH	7.4, 7.8, 8.1	TA, pH	20 d	27°C
Checkley *et al.* 2009 [307]	*Atractoscion nobilis*	P_{CO2}	380, 993, 2558	TA, DIC	7-8 d	18°C
Cripps *et al.* 2011 [319]	*Pseudochromis fuscus*	P_{CO2}	44-451, 607-630, 925-949	TA, pH	4-7 d	27-28°C
Dixson *et al.* 2010 [311]	*Amphiprion percula*	P_{CO2}	control?, 1050	pH	11 d	30°C
Dixson *et al.* 2014 [322]	*Mustelus canis*	P_{CO2}	405, 740, 1060	pH, TA	5 d	20°C

Continued on next page

Table S2 – continued from previous page

Reference	Species	Control variable	Expt.s	Measured carbon	Exposure time	Other detail
Domenici *et al.* 2012 [317]	*Neopomacentrus azysron*	P_{CO2}	441,880	TA, pH	4 d	28°C
Ferrari *et al.* 2011 [318]	*Pseudochromis fuscus*, *Pomacentrus moluccensis*, *P. amboinensis*, *P. nagasakiensis*, *P. chrysurus*	P_{CO2}	441, 718	TA, pH	4 d	28°C
Ferrari *et al.* 2012 [312]	*Pomacentrus amboinensis*	P_{CO2}	441, 554, 718, 880	TA, pH	4 d	27-28°C
Ferrari *et al.* 2012 [316]	*Pomacentrus amboinensis*	P_{CO2}	441, 718, 880	TA, pH	4 d	28°C
Jutfelt *et al.* 2013 [320]	*Gasterosteus aculeatus*	P_{CO2}	330, 991	TA, P_{CO2}	20-23, 40-43 d	18°C
Maneja *et al.* 2013 [308]	*Gadus morhua*	P_{CO2}	367, 1882, 4257	?	7-46 d	5-10°C
Maneja *et al.* 2013 [321]	*Gadus morhua*	P_{CO2}	359-373, 1810-1957, 4026-4170	TA, DIC	12, 27 d	5-10°C
Melzner *et al.* 2009 [295]	*Gadus morhua*	P_{CO2}	523-533, 3050, 5749	DIC, pH	4-12 min	5°C
Munday *et al.* 2009 [314]	*Amphiprion percula*	P_{CO2}	390, 1050, 1710	P_{CO2}	11 d	30°C
Munday *et al.* 2010 [313]	*Amphiprion percula*, *Pomacentrus wardi*	P_{CO2}	390, 541, 699, 863	P_{CO2}	1-10 d	28-30°C
Munday *et al.* 2011 [309]	*Amphiprion percula*	P_{CO2}	404, 1050, 1721	TA, pH	11 d	30°C
Nilsson *et al.* 2012 [323]	*Amphiprion percula*	P_{CO2}	452, 945	TA, pH	11 d	29°C
	Neopomacentrus azysron	P_{CO2}	440, 880	TA, pH	4 d	28°C
Simpson *et al.* 2011 [315]	*Amphiprion percula*	P_{CO2}	391, 613, 718, 876	TA, pH	17, 20, 24, 36 d	30°C

Fish – Development

Reference	Species	Control variable	Expt.s	Measured carbon	Exposure time	Other detail
Baumann *et al.* 2012 [306]	*Menidia beryllina*	P_{CO2}	Ex 1: 390, 1050<br>Ex 2: 390, 650, 810, 1070<br>Ex 3: 410, 780<br>Ex 4: 390, 890	DIC, pH	7 dph	24°C, O_2=8mg/L
Franke & Clemmesen 2011 [305]	*Clupea harengus*	P_{CO2}	480, 1260, 1859, 2626, 2903, 4635	pH	∼8 d	12°C, S=14, L:D=12:12
Frommel *et al.* 2010 [304]	*Gadus morhua*	P_{CO2}	380, 1400	pH	10 s	9.4°C
Frommel *et al.* 2012 [300]	*Gadus morhua*	P_{CO2}	380, 1800, 4200		7 w	2300-L mesocosms
Hurst *et al.* 2013 [303]	*Theragra chalcogramma*	P_{CO2}	Ex 1: 310, 475, 828, 1933<br>Ex 2: 287, 457, 805, 1773<br>Ex 3: 293, 411, 910, 1812<br>Ex 4: 297, 426, 941, 1858	DIC, TA	5-38 dph	8°C

Continued on next page

Table S2 – continued from previous page

Reference	Species	Control variable	Expt.s	Measured carbon	Exposure time	Other detail
Kikkawa *et al.* 2004 [301]	*Pagrus major*	pH<br>CO_2	5.9, 6.2<br>5, 10%	pH<br>CO_2	10-12 dph	20°C, O_2=20.95%
