# Supplementary material for: Effects of Ocean Acidification on Temperate Coastal Marine Ecosystems and Fisheries in the Northeast Pacific
Source: PLoS One. 2015 Feb 11;10(2):e0117533. doi: 10.1371/journal.pone.0117533 (PMC4324998; doi:10.1371/journal.pone.0117533)
Supplement: S3 Table — Details (e.g. species, location, and carbon state) for OA-related in situ studies of marine organisms. (PDF) [file pone.0117533.s003.pdf]

Table S3. Natural assemblages without manipulation – details for references in the paper. P_{CO2} is presented in units of μatm. Ω_{arag} is the aragonite saturation state: ($[Ca^{2+}]\cdot[CO_3^{2-}]$) / K_a, where K_a represents the solubility constant of aragonite a. The carbonate parameters measured within the seawater treatments (regardless of control variable) are reported. Other detail: S = salinity, I = irradiance, γ = photons, L:D = light:dark regime, T = temperature. n.r. = not reported or not measured

Reference	Species	Measured variable	In situ value	Measured carbon	Location	Other detail
**Macroalgae & Seagrass**						
Arnold *et al.* 2012 [140]	*Cymodocea nodosa*	P_{CO2}	422-4009	P_{CO2}, pH	Sicily	volcanic CO_2 vent, Island of Vulcano
Hendriks *et al.* 2014 [137]	*Posidonia oceanica*	pH	7.97-8.19	pH, TA	W Mediterranean, Spain	measurements: Sep 2011, Jun 2012
Johnson *et al.* 2012 [128]	*Patina avionic*	P_{CO2}	276-23,095	TA, pH	Mediterranean Sea and Papua New Guinea	Sea vents; range of *in situ* values shown for temperate waters of the Mediterranean Sea
**Zooplankton – Pteropods**						
Bednarsek *et al.* 2012 [172]	*Limacina helicina antarctica*	Ω_{arag}	~1.1-1.9 vs. ~0.997-2.2	DIC, TA	Southern Ocean	0-400 m depth
Bednarsek et al. 2014 [174]	*Limacina helicina*	Ω_{arag} (% water column undersaturated)	0-80%	DIC, TA w/ empirical model results	U.S. Pacific coast	0-200 m
Roger *et al.* 2012 [173]	*Creseis acicula*, *Daicavolinia longirostris*	Ω_{arag}	~3.65-3.9		North Australia	model hindcast, surface only, 1963-2009
**Zooplankton – Foraminifera**						
Moy *et al.* 2009 [145]	*Globegeria bulloides*	P_{CO2}	~195 - 280		Southern Ocean	sediment cores ~50,000-5,000 y before present
