# Supplementary material for: Effects of Ocean Acidification on Temperate Coastal Marine Ecosystems and Fisheries in the Northeast Pacific
Source: PLoS One. 2015 Feb 11;10(2):e0117533. doi: 10.1371/journal.pone.0117533 (PMC4324998; doi:10.1371/journal.pone.0117533)
Supplement: S1 Text — Marine animal use protocols in British Columbia, Canada. (PDF) [file pone.0117533.s004.pdf]

Pacific Region Animal Care Committee – Protocols

Intranet site: http://sci.info.pac.dfo.ca/pracc/Protocol-eng.htm

DFO Activities Requiring Animal Use Protocols

1. Holding of live vertebrates, cephalopods and other advanced invertebrates for research, testing or teaching, even for short periods (hours). This includes live displays and holding in tanks aboard ships, floating in natural waters etc. Objectives of the confinement should be stated clearly in the Protocol, together with a description of any interventions e.g. sampling;
2. Tagging of live animals that involves restraint and/or taking of measurements and/or tissue samples (includes most forms of tags e.g. plastic or metal insertions, cold/hot brands, and Visual Implant, radio, data-logging and PIT tags).
3. Lethal field sampling for research, teaching or non-routine testing.

EXEMPTIONS

1. Hatchery stock reared for release or destined for future use in research. Routine hatchery therapeutic practice and monitoring of growth is accepted under this exemption. Note that this exemption depends on ample precedent, e.g. the case of salmonids where culture technology is well-understood. If the species being cultivated lacks a broad base of previous knowledge of its culture and handling requirements, this exemption cannot be applied;
2. Mark-recapture activities with fish and invertebrates for abundance estimates and other parameters required purely for assessing stock;
3. Counting of fish at installations such as river fences and traps;
4. Lethal sampling of fish and invertebrates (e.g. gillnetting or trawling) for mandated monitoring of levels of disease, toxins and contaminants, or for Fish Inspection purposes, abundance estimates and other population parameters required purely for stock assessment;
5. Routine coded-wire tagging in general;
6. Sampling from commercial operations where animals are already dead or certain to die as a result of standard commercial practice;
7. Eggs or larvae that will not be allowed to reach first-feeding stage.
